# Hierarchical targeting biomimetic nanoparticles directing to lesion site and mitochondria promote neuroregeneration and attenuate inflammation after spinal cord injury

**DOI:** 10.1016/j.mtbio.2026.103544

**Published:** 2026-08-12

**Authors:** Yu Zhou, Yijie Kong, Zhiquan Yang, Kai Zhang, Runlin Wen, Shiyu Fu, Wanrong Ma, Ge Long, Xing Li, Dingyang Liu

**Affiliations:** a Department of Neurosurgery, Xiangya Hospital, Central South University, Changsha, Hunan, 410008, China; b Department of Biochemistry and Molecular Biology, School of Life Sciences, Central South University, Changsha, Hunan, 410078, China; c Department of Anesthesia, The Third Xiangya Hospital, Central South University, Changsha, Hunan Province, China

**Keywords:** Spinal cord injury, Biomimetic nanoparticle, Oxidative stress, SIRT3, Dihydromyricetin, Mitochondria

## Abstract

Following primary spinal cord injury (SCI), mitochondrial dysfunction induces oxidative stress, which in turn exacerbates secondary injury, including neuronal loss and the formation of an inhibitory microenvironment. To intervene in this pathological cascade, we constructed a hierarchically targeted biomimetic nanomedicine (MM@DHM) designed for sequential delivery to the lesion site and subsequently to mitochondria. Macrophage membrane coating mediates first-stage targeting to the injured spinal cord, while triphenylphosphonium (TPP) modification directs second-stage accumulation within mitochondria of lesional cells, enabling precise delivery of the SIRT3 agonist dihydromyricetin (DHM) to its site of action. Mechanistically, MM@DHM activates SIRT3, promotes SOD2 deacetylation, and enhances Pink1-Parkin-mediated mitophagy, consequently alleviating oxidative stress and restoring mitochondrial homeostasis. These integrated cellular effects confer robust neuroprotection, attenuate neuroinflammation, and improve functional recovery in a mouse model of SCI. Collectively, this work demonstrates that mitochondrial targeting represents a promising therapeutic strategy for ameliorating oxidative damage and mitochondrial dysfunction in SCI, and underscores the potential of cascade-targeted nanoplatforms for central nervous system repair.

## Graphical abstract

**Schematic illustration of MM@DHM for complete SCI therapy.** A cascade-targeting nanodrug, designated MM@DHM, was developed by coating DHM-loaded PLGA nanoparticles with TPP-modified macrophage membranes. This system successfully traverses the blood-spinal cord barrier and accumulates specifically at lesion sites and within mitochondria. The nanodrug operates by activating the mitochondrial SIRT3 signaling pathway, which concurrently mitigates ROS accumulation and promotes mitophagy. Consequently, it effectively suppresses the inflammatory response and stimulates axonal regeneration, leading to a substantial recovery of both motor and sensory functions.

**Figure:**
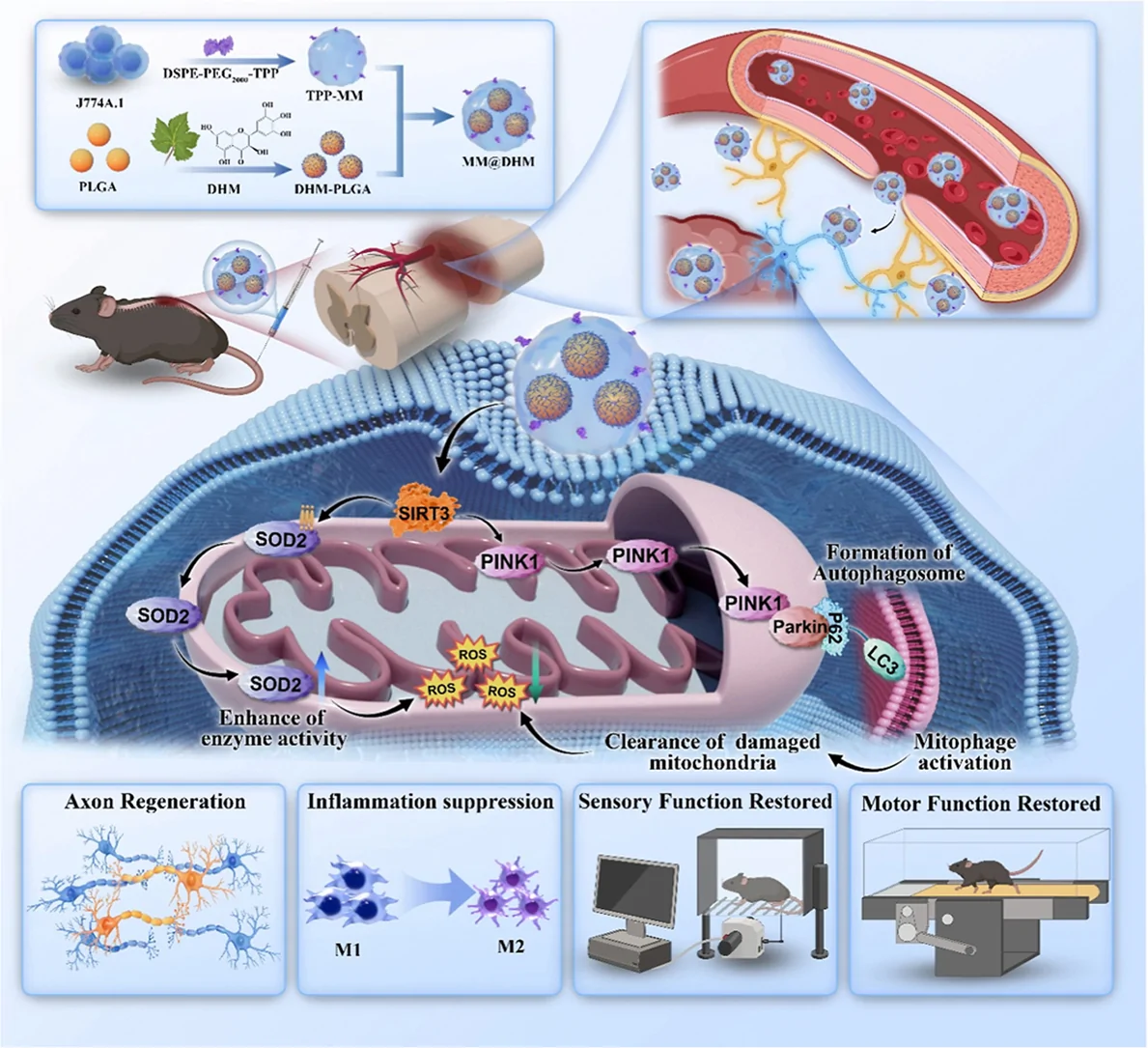

## 1 Background

Spinal cord injury (SCI) is a severe disorder of the central nervous system (CNS) that often leads to permanent loss of autonomic function and persistent sensory-motor deficits below the injury level [1,2]. The limited neurological recovery after CNS injury is mainly due to weak endogenous neurogenesis and a hostile microenvironment at the injury site, which contribute to neuronal loss and axonal degeneration [3,4]. Following the initial mechanical insult that causes axonal rupture and neural circuit disruption, oxidative stress becomes predominant during the acute and subacute phases. Reactive oxygen species (ROS) levels peak within hours to days post-injury. This dramatic rise in ROS exacerbates neuronal death, demyelination, and axonal degeneration, further worsening neurological impairment [5]. Additionally, oxidative stress promotes neuroinflammation, as high ROS levels enhance the activation of immune-related cells and stimulate pro-inflammatory cytokine release [6].

Moreover, excessive ROS induces mitochondrial dysfunction [7]. Mitochondria are essential in regulating calcium signaling, ATP production, apoptosis, cell growth, and more importantly, ROS generation [8]. The mitochondrial respiratory chain, or electron transport chain (ETC), is central to oxidative phosphorylation and ROS production. However, overaccumulation of ROS disrupts electron transfer, aggravating mitochondrial dysfunction [9]. This impairment reduces the energy supply necessary for neuronal integrity and function, while further increasing ROS production and causing oxidative damage to proteins, lipids, and DNA, thereby compromising neuronal activity [10]. During neural regeneration, mitochondrial dynamics—including biogenesis, transport, mitophagy, fusion, and fission—are key mechanisms in mitochondrial quality control [11,12]. Emerging strategies to alleviate mitochondrial dysfunction have focused on harnessing endogenous protective mechanisms, including the enhancement of mitophagy to remove damaged mitochondria, as well as anti-inflammatory and antioxidant responses. [[13], [14], [15], [16]]. These approaches have shown therapeutic potential in neurodegenerative diseases such as Parkinson's disease and Alzheimer's disease [17,18]. This endogenous anti-ROS system is largely orchestrated by SIRT3, a mitochondrial deacetylase that integrates mitophagy, antioxidant responses, and mitochondrial quality control.

Sirtuins are a family of NAD^+^-dependent protein deacetylases with seven members (SIRT1-7) in mammals, all exhibiting strong deacetylase activity [19]. SIRT3, which is primarily located in the mitochondrial matrix, plays a critical role in regulating mitochondrial metabolism, including the tricarboxylic acid cycle, fatty acid oxidation, oxidative phosphorylation, ROS detoxification, and mitochondrial dynamics [20,21]. Previous studies have shown that SIRT3 activation after myocardial injury reduces acetylation of SOD2, improves mitochondrial oxygen consumption, and inhibits ROS production [22]. Notably, SOD2 is a key mitochondrial antioxidant enzyme that neutralizes superoxide radicals, protecting cells from oxidative damage [23]. Furthermore, under conditions of severe mitochondrial damage, SIRT3 can deacetylate and activate FOXO3a to initiate mitophagy, clearing dysfunctional mitochondria [24]. To date, no highly specific and potent SIRT3 activator has been successfully developed. However, several small-molecule compounds with therapeutic potential have been reported. The natural flavonoid dihydromyricetin (DHM) has been identified as a potential SIRT3 agonist, shown to alleviate cartilage degeneration in osteoarthritis via SIRT3 activation [25]. Another study indicated that DHM improves diabetic cardiomyopathy by activating SIRT3 to suppress oxidative stress, inflammation, and necroptosis [26]. Despite its promise, DHM—like many natural compounds—has poor targeting ability, low water solubility, limited bioavailability, and a short half-life, which hinder its clinical application. Therefore, there is an urgent need to develop novel drug delivery systems with improved mitochondrial targeting capability for SCI treatment.

Bionic nanoparticle delivery systems have attracted growing interest in recent years [27,28]. By inheriting biological functions from source cell membranes, biomimetic nanocarriers offer advantages such as high targeting specificity, excellent biocompatibility, immune evasion [[29], [30], [31]], and extended circulation half-life [32]. Macrophages are the primary immune effector cells after SCI and play a central role in the inflammatory cascade during secondary injury [33]. Previous studies have shown that macrophage membrane-coated nanoparticles exhibit strong inflammatory tropism and efficient drug-loading capacity. Interactions between integrins (e.g. integrin-α4) on macrophage membranes and vascular cell adhesion molecule-1 (VCAM-1) on damaged endothelial cells facilitate nanoparticle penetration across the blood-brain barrier (BBB) into inflammatory sites [34]. Owing to their straightforward preparation and preserved membrane functionality, macrophage membrane-based biomimetic nanoparticles have emerged as effective drug carriers for neurological disorders [35,36]. However, conventional macrophage-mimetic nanoparticles lack intrinsic mitochondrial targeting ability, which restricts drug accumulation within mitochondria. Further modification is thus necessary. Mitochondria exhibit a high transmembrane potential across the inner membrane, which promotes selective accumulation of cationic molecules such as triphenylphosphonium (TPP) through electrostatic interactions [37]. Because of its mitochondrial-targeting properties, TPP has been widely used to deliver probes, antioxidants, and drugs to mitochondria [[38], [39], [40]]. Compared to other mitochondrial targeting strategies (e.g., those based on lipid composition or specific signaling), TPP offers a more efficient, versatile, and reliable approach [41,42].

In this study, we developed a mitochondria-targeted nanodrug for SCI treatment using TPP-modified macrophage membrane-coated poly (lactic-co-glycolic acid) (PLGA) nanoparticles loaded with DHM. The macrophage membrane coating enables the nanodrug to cross the BBB and accumulate at the lesion site via inflammatory chemotaxis after intravenous administration. TPP modification on the membrane enhances cellular uptake and promotes mitochondria-specific drug delivery. In vitro experiments showed that the nanodrug possesses excellent antioxidant properties, reducing ROS levels and oxidative stress in HT-22 neuronal cells under oxidative damage, while promoting mitophagy and improving mitochondrial function. It also attenuated oxidative stress-induced neuronal death and supported axonal regeneration. Notably, the nanoparticles prompted a shift from pro-inflammatory to anti-inflammatory phenotypes in oxidatively stressed BV2 microglial cells. In vivo studies similarly indicated substantial neuroprotective and anti-inflammatory effects. Transcriptomic analysis revealed that nanodrug treatment significantly upregulated genes related to mitochondrial function, oxidative stress response, and neural regeneration. Behavioral tests in SCI models confirmed that the nanodrug promoted sensory-motor functional recovery. These findings provide preliminary evidence that our mitochondria-targeted biomimetic nano-delivery system simultaneously supports axonal regeneration and modulates neuroinflammation. This dual-target therapeutic strategy may offer a promising avenue for effective treatment of SCI.

## 2 Methods

### Ethical approval

All animal experiments were conducted in strict accordance with the National Institutes of Health (NIH) Guide for the Care and Use of Laboratory Animals and were approved by the Animal Care and Use Committee of Xiangya Hospital, Central South University (Approval No.: CSU-2024-0258).

### 2.1 Establishment of animal models

A total of 134 female C57BL/6J mice (6 weeks old) were obtained from Slac Animals (Changsha, China). All procedures were conducted in accordance with the ethical guidelines set forth in the Guide for the Care and Use of Laboratory Animals (National Institutes of Health, USA). Mice were randomly allocated to different treatment groups, receiving intravenous injections of various nanomedicines post-injury, and were housed under controlled conditions with a 12-h light/dark cycle. The specific experimental groupings and animal treatments were as follows:

A MM@DHM group (n = 33):

(1) Spinal cord injury (SCI) model establishment: After intraperitoneal anesthesia with sodium pentobarbital (100 mg/kg), a complete spinal cord transection model was created. A midline dorsal incision was made to expose the surgical field at the T8 vertebral level, followed by laminectomy of approximately 3 mm at T8 and resection of a 1.5-mm segment of spinal cord tissue. Hemostasis was achieved using medical gelatin sponges, and the muscle and skin layers were sutured in sequence.

(2) MM@DHM administration: At 5 min post-SCI, MM@DHM was administered via tail vein injecton at a dose of 40 mg/kg (dispersed in normal salinez, equivalent to a DHM dose of 2 mg/kg). B.

B MM@PLGA group (n = 29):
(1) SCI model establishment: Same as above.
(2) MM@PLGA administration: At 5 min post-SCI, MM@PLGA was given via tail vein injection at a dose of 40 mg/kg (dispersed in normal saline).
C PLGA group (n = 9):
(1) SCI model establishment: Same as above.
(2) PLGA administration: At 5 min post-SCI, Cy5.5-labeled PLGA was administered via tail vein injection at a dose of 40 mg/kg (dispersed in normal saline).
D DHM group (n = 23):
(1) SCI model establishment: Same as above.
(2) DHM administration: At 5 min post-SCI, DHM was injected via the tail vein at a dose of 2 mg/kg (dispersed in normal saline).
E SCI group (n = 32):
(1) SCI model establishment: Same as above.
(2) Vehicle administration: At 5 min post-SCI, normal saline (0.15 mL per mouse, approximately equivalent to the volume of previous injections) was administered via tail vein injection.

F. Sham group (n = 8): After intraperitoneal anesthesia with sodium pentobarbital (100 mg/kg), a sham operation was performed. A midline dorsal incision was made to expose the T8 vertebral level, and a laminectomy of approximately 3 mm at T8 was carried out without damaging the spinal cord tissue. Hemostasis was achieved using medical gelatin sponges, and the muscle and skin layers were sutured in sequence.

### 2.2 Isolation of neurons

Neonatal mice (1-2 days old) were purchased from Slac Animals (Changsha, China). After decapitation, bilateral telencephalons were extracted. Under a dissecting microscope, meninges were carefully removed, and cortical brain tissue was dissected and minced using micro-scissors. The tissue suspension was digested with 0.25% trypsin for 20 min, filtered through a 100-μm cell strainer, and centrifuged. The pellet was resuspended in neuronal basal medium supplemented with 2% B27, 1% glutamine, and 1% penicillin/streptomycin. Cells were counted and seeded at a density of 5 × 10^5 cells/well in 6-well plates. After 3 days of culture, interventions were applied, and axonal length was measured via immunofluorescence staining at day 5.

### 2.3 Preparation of MM@DHM

First, 100 mg PLGA and 2 mg CY5.5 were co-dissolved in 2.4 mL dichloromethane, while 5 mg DHM was dissolved in 1 mL methanol. The solutions were added to 20 mL of 5% (w/v) polyvinyl alcohol aqueous solution and emulsified by sonication for 10 min. The mixture was stirred for 1 h to remove dichloromethane and methanol, then centrifuged at 13,300 rpm for 5-10 min. The pellet was washed twice with water and resuspended in 10 mL water to obtain PLGA nanoparticles loaded with DHM and CY5.5. Next, thawed J774A.1 cells were centrifuged at 2000 rpm for 5 min to collect the pellet. The pellet was resuspended in phosphate buffer saline (PBS) with protease inhibitors, homogenized, and centrifuged at 2000 rpm for 5 min to collect the supernatant. The remaining pellet was re-homogenized and centrifuged at 12,000 rpm for 20 min to collect the membrane fraction. The protein concentration of the combined membrane fractions was quantified. The PLGA nanoparticles loaded with DHM and CY5.5 were mixed with J774A.1 cell membranes at a 1:1 vol ratio, followed by incubation with 2 mg of DSPE-PEG-TPP at 37 °C for 30 min. The mixture was then extruded through a liposome extruder equipped with a polycarbonate membrane (pore size: 100 nm) to obtain MM@DHM. The resulting nanoparticles were characterized as described in the Results section (3.2).

### 2.4 Immunofluorescence

For tissue immunofluorescence staining, post-injury mice were perfused with 4% paraformaldehyde, and the spinal cords were dissected and fixed overnight in 4% paraformaldehyde. The tissues were dehydrated in 20% sucrose for 1 day, followed by 30% sucrose for 3 days at 4 °C. The samples were embedded, and 13-μm-thick sections were cut along the dorsoventral axis using a cryostat (RWD Life Science, Shenzhen, China). The sections were washed three times with PBS for 5 min each, permeabilized with 5% Triton X-100 for 15 min, and blocked with 5% BSA for 30 min. Primary antibodies, including anti-Tuj-1 (abcam, ab18207), anti-GFAP (abcam, ab7260), anti-MBP (abclone, A25814), anti-SMA (proteintech, 67735-1-lg), anti-IBA-1 (oasis biofarm, OB-PGP049), anti-INOS (abcam, ab15323), anti-CD206 (proteintech, 18704-1-AP), and anti-SYN (abclone, A24122), were applied. After incubation, the sections were washed three times with PBS for 5 min each and incubated with secondary antibodies, for 2 h at room temperature. The sections were washed three times with PBS for 5 min each, mounted with antifade mounting medium containing DAPI, and observed under a fluorescence microscope (ZEISS, Axio Imager M2, Germany).

### 2.5 H&E staining

Spinal cord cryosections were stained with hematoxylin for 7 min, rinsed with tap water, differentiated for 2 s, blued for 1 min, and counterstained with eosin for 2 min. The sections were dehydrated through three changes of 100% alcohol (1 min each), cleared in xylene, and mounted with neutral balsam for microscopic examination.

### 2.6 TUNEL staining

Apoptosis in spinal cord tissue was detected using a TUNEL staining kit (Roche, No. 12156792910) according to the manufacturer's instructions. The sections were incubated with the TUNEL reaction mixture for 60 min at 37 °C, followed by DAPI staining to label nuclei.

### 2.7 TEM

Cells subjected to different treatments were scraped and fixed in 2.5% glutaraldehyde for 2 h, rinsed with 0.1 M phosphate buffer, and post-fixed in 1% osmium tetroxide. The samples were dehydrated through a graded series of ethanol and acetone, embedded, and polymerized. Ultrathin sections were stained with 2% uranyl acetate and lead citrate and observed under a transmission electron microscope (HT7800, 80 kV).

### 2.8 Western blotting

Cells or spinal cord tissue were homogenized in cold RIPA buffer (P101, Epizyme Biotech) supplemented with protease and phosphatase inhibitors (GRF101, 102, Epizyme Biotech). The homogenates were incubated on ice for 30 min and centrifuged at 15,000 × g for 20 min at 4 °C to remove debris. Protein concentration was determined using a BCA protein assay kit (ZJ102, Epizyme Biotech). The lysates were diluted with 4× loading buffer (LT-101S, Epizyme Biotech), boiled at 95 °C for 5 min, and separated on 10% SDS-PAGE gels. The proteins were transferred to PVDF membranes, blocked with 5% skim milk in TBST for 60 min at room temperature, and incubated with primary antibodies diluted in 3% BSA overnight at 4 °C on a shaker. After six washes with TBST (10 min each), the membranes were incubated with secondary antibodies in 5% milk for 30 min at room temperature on a shaker, followed by three TBST washes before development.

### 2.9 Enzyme-linked immunosorbent assay (ELISA)

Spinal cord tissue homogenates were prepared as described in section 2.9. The levels of INOS and CD206 in tissue homogenates were quantified using commercial mouse ELISA kits (INOS: ELK1504, CD206/MRC1: ELK3454, ELK Biotechnology) according to the manufacturer's instructions. Briefly, 100 μL of serially diluted standard working solutions or samples were added to each well and incubated at 37 °C for 80 min. After removing the liquid, the wells were washed three times with 200 μL of wash buffer. Then, 100 μL of biotinylated antibody working solution was added to each well and incubated at 37 °C for 50 min. Following three washes, 100 μL of HRP enzyme working solution was added and incubated at 37 °C for 50 min. After five washes, 90 μL of TMB substrate was added and incubated at 37 °C for 20 min in the dark. Finally, 50 μL of stop solution was added, and the optical density was immediately measured at 450 nm using a microplate reader. Cytokine concentrations were calculated from the standard curve and then normalized to the total protein concentration of each sample.

### 2.10 In vitro oxidative stress models and drug treatments

To establish the in vitro oxidative stress model, HT-22 cells and BV2 cells were treated with 100 μM H2O2, while primary cortical neurons were treated with 20 μM H_2_O_2_, all for 24 h at 37 °C. For drug treatment groups, cells were incubated with free DHM (7 μg/mL) or MM@DHM (140 μg/mL, equivalent to 7 μg/mL DHM based on a drug loading of 5.23%), followed by co-incubation with H2O2 at the respective concentrations for 24 h.

For BBB penetration assays (Section 3.2), BV2 cells were activated with H_2_O_2_ for 12 h prior to co-culture with bEnd.3 monolayers in Transwell inserts. The nanoparticles (MM@DHM, MM@PLGA or PLGA, at the same concentration as above) were added to the upper Transwell chamber and incubated for 24 h. For mitochondrial colocalization experiments, the nanoparticles were directly added to the cell culture medium at the same concentration (include “without TPP”) and incubated with the cells. For other experiments involving BV2 microglial polarization assessment (immunofluorescence, flow cytometry, and RT-qPCR), cells were treated with the same drug concentrations as described above.

### 2.11 ROS detection in vitro

For cellular ROS detection, HT22 cells treated with different interventions were washed with cold PBS and incubated with H2DCFDA working solution (MedChemExpress, HY-D0940) for 30 min at 37 °C. The cells were immediately washed with PBS, and ROS levels were assessed by fluorescence microscopy.

### 2.12 Mitochondrial function assays

Mitochondrial reactive oxygen species (mitoROS) levels were measured using MitoSOX Red (MedChemExpress, HY-D1055). After the indicated treatments, HT-22 cells were washed twice with cold PBS and then incubated with MitoSOX Red working solution at 37 °C for 30 min in the dark. Cells were washed three times with cold PBS and immediately visualized under a fluorescence microscope.

Mitochondrial membrane potential was assessed using the JC-1 fluorescent probe (MedChemExpress, HY-15534). Cells were washed twice with cold PBS and incubated with JC-1 working solution at 37 °C for 30 min in the dark. After three washes with cold PBS, cells were immediately imaged. The ratio of red (aggregate) to green (monomer) fluorescence intensity was calculated to evaluate changes in membrane potential.

For mitophagy assessment, HT-22 cells were co-stained with MitoTracker (to label mitochondria) and an antibody against LC3B (Bioswamp Life Science Lab, Wuhan, China, RMAB54400), a canonical autophagosome marker. After incubation with MitoTracker according to the manufacturer's instructions, cells were fixed, permeabilized, and immunostained for LC3B. The percentage of MitoTracker-positive position that co-localized with LC3B was quantified to evaluate mitophagic activity.

### 2.13 Live/dead staining

HT-22 cells were seeded in 24-well plates at 2*10^4^ cells per well. After the indicated treatments, cells were washed twice with PBS and stained with a Calcein-AM/PI double staining kit (Beyotime, C2015) according to the manufacturer s instructions. Briefly, cells were incubated with 2 μM Calcein-AM and 4 μM PI in PBS for 30 min at 37 °C in the dark. Cells were washed twice with PBS and immediately imaged under a fluorescence microscope.

### 2.14 BBB penetration assay

The in vitro blood-brain barrier (BBB) permeability assay was performed using a Transwell co-culture system. Briefly, bEnd.3 cells were seeded on collagen-coated Transwell inserts until form a monocell layer. BV2 cells (resting or H2O2-activated) were seeded in the lower chamber. MM@DHM was added to the upper chamber and incubated for 24 h at 37 °C. The medium in the lower chamber was collected, and the fluorescence intensity of Cy5.5 (excitation/emission: 650/718 nm) was measured using a microplate reader to quantify the amount of nanoparticles that crossed the bEnd.3 monolayer.

### 2.15 Flow cytometry

For microglial polarization analysis, BV2 cells were collected after the indicated treatments, washed with PBS, and resuspended at 1 × 10^6^ cells/mL. For simultaneous surface and intracellular staining, cells were first blocked with FcR blocking reagent for 10 min at 4 °C, then incubated with rabbit anti-CD206 primary antibody (Proteintech, 18704-1-AP, 1:200) for 30 min at 4 °C in the dark. After washing, cells were incubated with Alexa Fluor 488-conjugated secondary antibody (1:500) for 30 min at 4 °C in the dark. Cells were then fixed with 4% paraformaldehyde for 15 min at room temperature, permeabilized with 0.1% Triton X-100 for 15 min at room temperature, and incubated with rat anti-INOS primary antibody (Invitrogen, MA5-42837, 1:100) for 30 min at 4 °C in the dark, followed by Alexa Fluor 568-conjugated secondary antibody (1:500) for 30 min at 4 °C in the dark. Cells were washed with PBS between each step. Finally, stained cells were resuspended in 200 μL of PBS containing 1% BSA and analyzed using a flow cytometer. Isotype controls and fluorescence-minus-one (FMO) controls were included for gating.

For JC-1 analysis, HT-22 cells were incubated with JC-1 working solution (5 μg/mL) at 37 °C for 20 min in the dark, washed, and immediately analyzed. The results were expressed as the ratio of green (monomer) fluorescence.

For live/dead cell analysis, HT-22 cells were stained with Calcein-AM (2 μM) and propidium iodide (4 μM) at 37 °C for 20 min in the dark and analyzed within 1 h. Live cells (Calcein-AM^+^, green) and dead cells (PI^+^, red) were quantified from at least 10,000 events per sample.

### 2.16 Motor function and pain sensitivity assessment

Hindlimb motor function was evaluated weekly for 10 WPI using the BMS by two independent observers. Motor coordination was assessed using a rotarod apparatus (YLS-4C), and the latency to fall was recorded. Footprint analysis was performed by coating the hind paws with dye and measuring stride length on a straight runway. Mechanical pain thresholds were evaluated using calibrated Von Frey filaments.

### 2.17 MEP recording

Under anesthesia, the scalp and hindlimbs were shaved and disinfected. The scalp was incised, and a cranial burr hole was made to insert the stimulating electrode. The sciatic nerve was exposed for placement of the recording electrode, and MEPs were recorded.

### 2.18 In vivo imaging for nano particles

Cy5.5-labeled PLGA, MM@PLGA, and MM@DHM were administered via tail vein injection. Fluorescence distribution was monitored at 1, 3, and 7 DPI using an IVIS imaging system (PerkinElmer, Waltham, MA, USA). After euthanasia, the heart, liver, spleen, lungs, kidneys, and spinal cord were excised for ex vivo imaging. Cy5.5 fluorescence signals were analyzed using Living Image software (PerkinElmer).

### 2.19 ROS detection in vivo

L012 sodium salt (MedChemExpress, HY-108537), a luminol-based chemiluminescent probe, was used to detect NADPH oxidase-derived superoxide. Mice received intraperitoneal injections of 25 mg/kg L012 at 1 and 3 DPI, and chemiluminescence was imaged 5 min later using the IVIS system.

### 2.20 In vivo toxicity evaluation

At 7 DPI, serum was collected to measure ALT, AST, UA, and BUN levels for assessing hepatorenal toxicity. Major organs, including the heart, liver, spleen, lungs, and kidneys, were subjected to H&E staining for histopathological evaluation.

### 2.21 RNA extraction and RT-qPCR

RNA was extracted via the RNA Mini Kit (Takara, Code No. 9767) according to the manufacturer's protocol, and cDNA was synthesized via the PrimeScript RT Reagent Kit (Takara, Catalog No. RR037A). To quantify the expression of INOS (5′-GAGACAGGGAAGTCTGAAGCAC-3′ and 5′-CCAGCAGTAGTTGCTCCTCTTC-3′), CD206 (5′-GTTCACCTGGAGTGATGGTTCTC-3′ and reverse 5′-AGGACATGCCAGGGTCACCTTT-3′), IL-1β (5′-TGGACCTTCCAGGATGAGGACA-3′) and Arg-1 (forward 5′-CATTGGCTTGCGAGACGTAGAC-3′ and reverse 5′-GCTGAAGGTCTCTTCCATCACC-3′) in each group, quantitative real-time PCR (RT-qPCR) was performed with TB Green (Takara, Code No. RR430) for amplification and detection. GAPDH (forward 5′-CCTGCGACTTCAACAGCAAC-3′ and reverse 5′-TGGGATAGGGCCTCTCTTGC-3′) was used as the internal control. The PCRs were carried out on a fluorescence quantitative PCR instrument (Bio-Rad), and the data were analyzed via the ΔΔCt method to determine gene expression levels.

### 2.22 RNA-seq and bioinformatics analysis

Spinal cord tissue within 1 mm of the injury site was collected, and total RNA was extracted using TRIzol (Invitrogen, CA, USA). RNA concentration (>50 ng/μL), RIN value (>7.0), and OD260/280 ratio (>1.8) were verified using a NanoDrop ND-1000 (NanoDrop, Wilmington, DE, USA) and Bioanalyzer 2100 (Agilent, CA, USA). mRNA was enriched using oligo(dT) magnetic beads (Dynabeads Oligo (dT), Thermo Fisher, USA), fragmented for 5-7 min at 94 °C using a magnesium RNA fragmentation module (NEBNext®, E6150S, USA), and reverse-transcribed into cDNA. Second-strand synthesis incorporated dUTP (Thermo Fisher, R0133, CA, USA). After end repair, A-tailing, and adapter ligation, libraries with insert sizes of 300 ± 50 bp were constructed and sequenced on an Illumina NovaSeq 6000 platform (LC Bio Technology CO., Ltd., Hangzhou, China) using PE150 chemistry. Raw reads were quality-controlled using fastp (https://github.com/OpenGene/fastp), aligned to the GRCh38 genome using HISAT2 (https://ccb.jhu.edu/software/hisat2), and quantified using StringTie (https://ccb.jhu.edu/software/hisat2) in FPKM units. Differential gene expression analysis was performed using edgeR (p < 0.05), and GO enrichment analysis was conducted using DAVID (https://david.ncifcrf.gov/).

### 2.23 Statistical analysis

Results are expressed as mean ± SD. All analyses were conducted using GraphPad Prism 9.0. Multiple group comparisons were performed by repeated-measures ANOVA (significance level α = 0.05), with Tukey's post hoc test for multiple comparisons. Two-group comparisons used two-tailed Student's t-tests. Quantitative immunofluorescence data were analyzed by ANOVA with Tukey's test, with statistical significance set at p < 0.05; BMS scores were compared using non-parametric tests (Mann-Whitney *U* test).

## 3 Results

### 3.1 SIRT3-mediated ROS Scavenging Potential of DHM

**Fig. 1:**
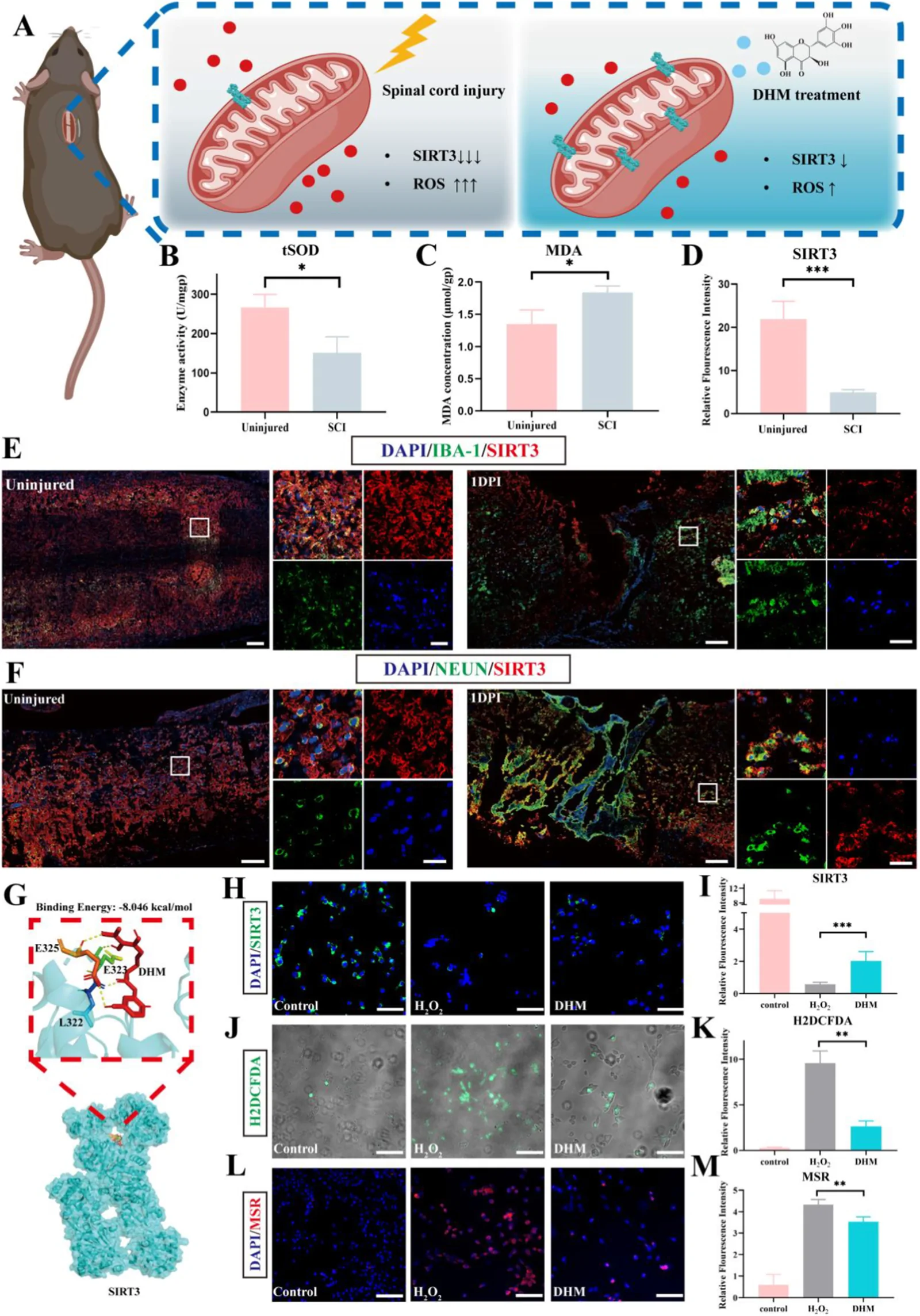
SIRT3-mediated ROS Scavenging Potential of DHM. (A) Schematic diagram illustrating the therapeutic mechanism of DHM in SCI via activation of mitochondrial SIRT3; (B) Effect of spinal cord injury on total superoxide dismutase (tSOD) activity; (C) Effect of spinal cord injury on malondialdehyde (MDA) concentration; (D) Quantification of SIRT3 fluorescence intensity in the lesion area of uninjured and 1-day post-spinal cord injury groups (n = 3 in B-D); (E) Differential expression levels of SIRT3 protein in IBA-1^+^ microglia within the lesion area between uninjured and 1-day post-spinal cord injury groups (scale bar: 200 μm; 20 μm); (F) Differential expression levels of SIRT3 protein in NEUN^+^ neurons within the lesion area between uninjured and 1-day post-spinal cord injury groups (scale bar: 200 μm; 20 μm); (G) Molecular docking of the DHM-SIRT3 complex. (H) Representative SIRT3 immunofluorescence staining of HT22 cells from different treatment groups. (scale bar: 100 μm); (I) Quantification of SIRT3^+^ signal from different treatment groups; (J) Representative fluorescence microscopy images of HT 22 cells stained with H_2_DCFDA from different treatment groups. (scale bar: 100 μm); (K) Quantification of H_2_DCFDA ^+^ signal from different treatment groups; (L) Representative fluorescence microscopy images of HT 22 cells stained with MitoSox Red (MSR) from different treatment groups. (scale bar: 100 μm); (M) Quantification of MSR^+^ signal from different treatment groups (n = 5 in H-M). Data shown in B-D, I, K, M are presented as the mean ± standard error of the mean. ***P < 0.001, **p < 0.01, *p < 0.05. (For interpretation of the references to colour in this figure legend, the reader is referred to the Web version of this article.)

The regulation of mitochondrial homeostasis and oxidative stress responses plays a crucial role in neuroprotection following SCI [43,44]. Our results demonstrate this by showing a significant decrease in total SOD activity alongside a significant increase in malondialdehyde concentration at 1 day post-injury (Fig. 1A–C). Moreover, previous studies have demonstrated that SIRT3 is critically involved in maintaining mitochondrial homeostasis and modulating oxidative stress responses [19,45]. To examine the cellular localization of SIRT3 after SCI, we performed immunofluorescence staining on spinal cord tissues at 1 day post-injury (DPI). Compared with the uninjured group, SIRT3 fluorescence intensity in the lesion area decreased by approximately 77.09% (Fig. 1 D), indicating a strong correlation between reduced SIRT3 expression and SCI. We next investigated which cell types express SIRT3 in the injured spinal cord. Co-staining with the pan-microglial marker IBA-1 and the neuronal marker NEUN revealed substantial co-localization of SIRT3 with both microglia/macrophages and neurons (Fig. 1E and F; Supplementary Figure S1 A-B). This observation confirms SIRT3 expression in microglia/macrophages, providing the rationale for our subsequent in vitro studies using BV2 microglial cells to explore the functional role of SIRT3 in these cells. Additionally, we assessed SIRT3 co-localization with INOS (an M1 marker) and CD206 (an M2 marker) at 1 DPI; as shown in Supplementary Figure S1 C-D, SIRT3 was found to be present in both pro-inflammatory and anti-inflammatory microglial phenotypes. Collectively, these findings suggest that SIRT3 is widely expressed in both neurons and microglia within the injured spinal cord, supporting the rationale for activating SIRT3 to modulate mitochondrial homeostasis and oxidative stress in these cell types as a potential therapeutic strategy for SCI.

DHM, a natural flavonoid containing multiple phenolic hydroxyl groups, has been identified as a potential SIRT3 agonist [25,46]. Molecular docking analysis revealed a strong binding affinity between DHM and SIRT3, with predicted interaction sites at E323, E323, and L322, and a binding energy of −8.046 kcal mol^−1^ (Fig. 1 G). These results suggest favorable binding compatibility between DHM and SIRT3.And our preliminary experimental results also indicate that the application of DHM can increase the expression level of SIRT3 in HT22 cells under H_2_O_2_ stimulation. Furthermore, DHM treatment similarly reduces both the overall ROS level and mitochondrial ROS production in HT22 cells, demonstrating its role in activating SIRT3 and counteracting ROS (Fig. 1H–M).

### 3.2 Synthesis and characterization of MM@DHM

**Fig. 2:**
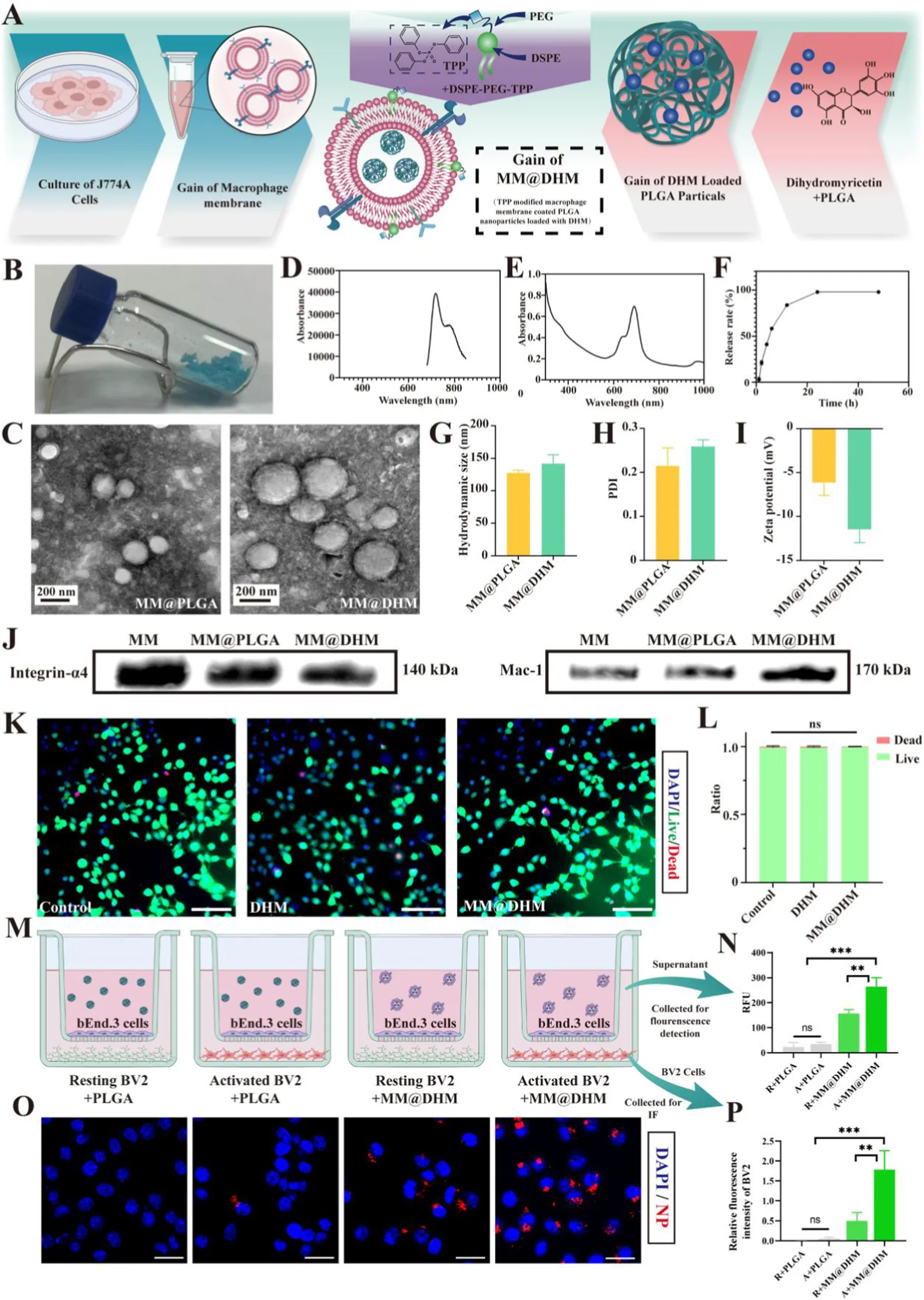
Synthesis and characterization of MM@DHM. (A) Schematic illustration of the preparation process of MM@DHM; (B) Gross photographs of MM@DHM; (C) Representative TEM image of MM@PLGA and MM@DHM; (D) Fluorescence spectra of MM@DHM; (E) UV-vis absorbance spectra of MM@DHM; (F) Drug release profile of MM@DHM; (G) Measurement of particle size of MM@PLGA and MM@DHM; (H) Measurement of polydispersity index of MM@PLGA and MM@DHM; (I) Measurement of zeta potential of MM@PLGA and MM@DHM, (n = 3 in G-I); (J) Western blot analysis of integrin-α4 and Mac-1 in macrophage membrane (MM), MM@PLGA and MM@DHM. (K) Live/dead staining of HT-22 cells after co-incubation with DHM or MM@DHM (scale bar: 100 μm). (L) Quantification of the ratio of live/dead cells from different treatment groups (n = 5). (M) Schematic of the in vitro BBB model. (N) Quantification of NP fluorescence in the lower receiver chamber. (O) Representative IF images of NP uptake by BV2 (scale bar: 25 μm). (P) Quantification of relative fluorescence in BV2 (n = 3 in N-P). Data shown in L, N, P are presented as the mean ± standard error of the mean. ***P < 0.001, **p < 0.01, and ns indicates no significance.

To achieve precise targeted drug delivery, we developed a multi-targeting nanoparticle system designated as MM@DHM. In brief, DHM-loaded PLGA nanoparticles were prepared and subsequently coated with macrophage membranes and DSPE-PEG-TPP via liposome extrusion to obtain MM@DHM (Fig. 2A and B). Transmission electron microscopy revealed well-defined spherical morphology for both MM@PLGA and MM@DHM (Fig. 2C). Fluorescence spectroscopy confirmed successful CY5.5 labeling with maximum emission at 718 nm (Fig. 2 D), while UV-Vis spectroscopy revealed strong absorption centered at 680 nm (Fig. 2 E). In vitro drug release studies demonstrated favorable release kinetics, with complete payload release achieved within 24 h (Fig. 2 F). Dynamic light scattering measurements showed that MM@DHM had an average particle size of 141.94 ± 13.50 nm, slightly larger than that of MM@PLGA (121.48 ± 4.15 nm) (Fig. 2 G). The polydispersity index was 0.26 ± 0.01 for MM@DHM and 0.22 ± 0.04 for MM@PLGA (Fig. 2H). And zeta potential measurements revealed values of −11.47 ± 1.49 mV for MM@DHM and −6.12 ± 1.50 mV for MM@PLGA (Fig. 2 I). The encapsulation efficiency (EE%) of DHM in MM@DHM was 55.20% ± 4.6%, with a drug loading (DL%) of 5.23% ± 0.4% (n = 3). These values were used to calculate the equivalent DHM concentration in all subsequent in vitro and in vivo experiments. Importantly, western blot analysis confirmed that the macrophage membrane coating effectively preserved key functional proteins, as evidenced by the retention of integrin-α4 and Mac-1 on MM@DHM, similar to the native macrophage membrane (Fig. 2 J). Finally, we evaluated the biocompatibility of the nanodrug by co-incubating HT-22 cells with either free DHM or MM@DHM for one day, followed by live/dead staining. Results show that MM@DHM did not induce additional cell death compared to free DHM, demonstrating its favorable biocompatibility (Fig. 2K and L).

Furthermore, we evaluated the BBB penetration ability of MM@DHM using a Transwell co-culture model. Resting or activated BV2 cells were seeded in the upper chamber and co-cultured with bEnd.3 monolayers. The results indicated that the trans-BBB transport of MM@DHM was significantly enhanced only when co-cultured with activated BV2 cells (Fig. 2M and N); meanwhile, immunofluorescence staining confirmed that the cellular uptake of MM@DHM by activated BV2 was significantly higher than that by resting BV2 (Fig. 2O and P). These findings collectively demonstrate that MM@DHM possesses excellent in vitro BBB penetration ability and can achieve specific accumulation at inflammatory lesions via inflammatory chemotaxis.

### 3.3 Lesion-targeting capability and biocompatibility of MM@DHM

**Fig. 3:**
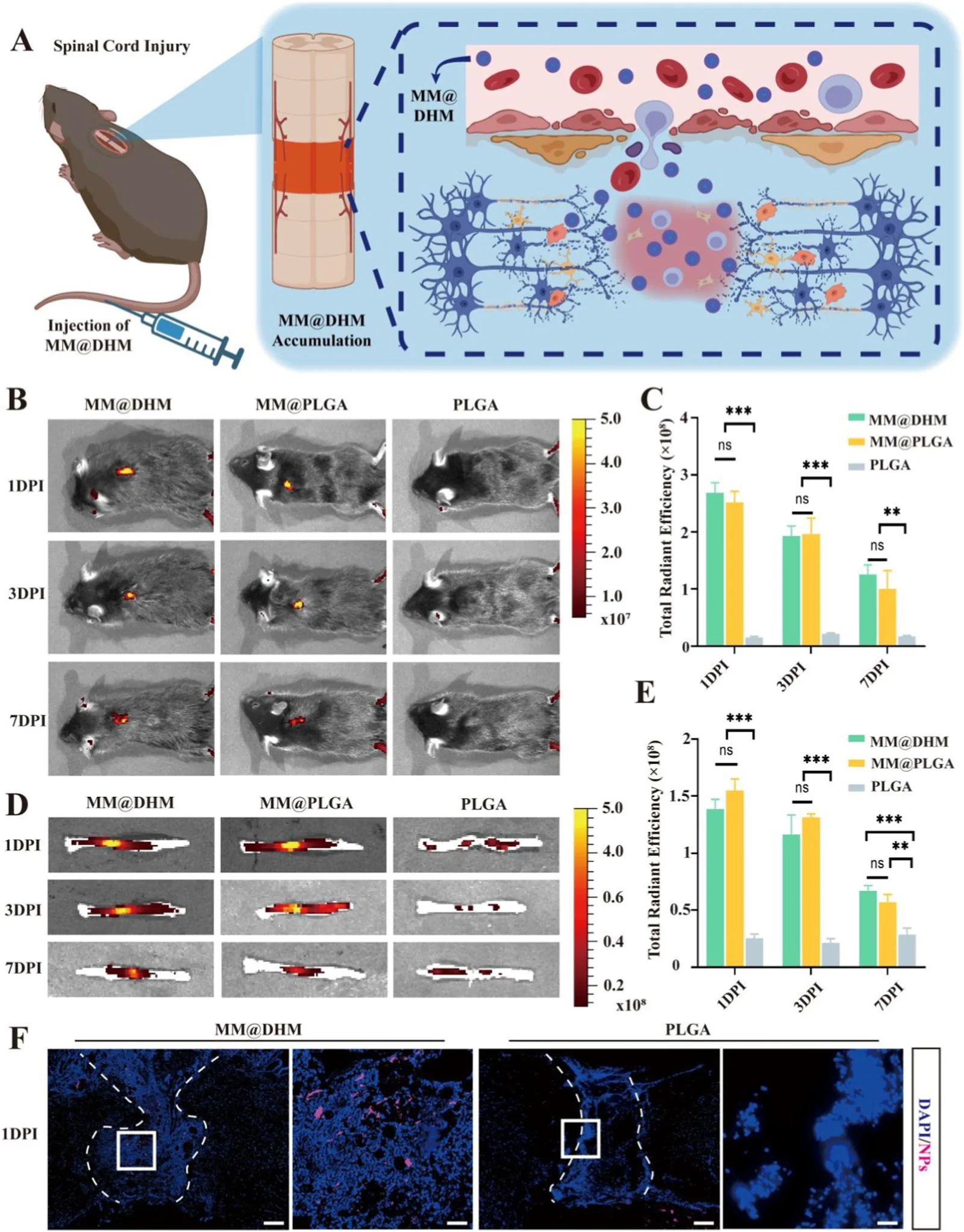
Lesion-targeting performance of MM@DHM. (A) Schematic illustration of intravenously injected MM@DHM crossing the blood-spinal cord barrier and targeting the lesion area; (B) Representative in vivo fluorescence images of SCI mice after intravenous injection with Cy5-labeled MM@DHM, MM@PLGA or PLGA; (C) Quantification of total radiant efficiency ([p]·[s]^−1^·[μW]^−1^·[cm^2^]) from different treated groups at different time points (n = 3); (D) Representative ex vivo fluorescence images of spinal cord tissues dissected from SCI model mice after intravenous injection with Cy5-labeled MM@DHM, MM@PLGA or PLGA; (E) Quantification of total radiant efficiency ([p]·[s]^−1^·[μW]^−1^·[cm^2^]) from different treated groups at different time points (n = 3); (F) Representative spinal cord fluorescence images showing MM@DHM accumulating at lesion site of 1DPI mice. (scale bar: 200 μm; 40 μm) Data shown in C and E are presented as the mean ± standard error of the mean. ***P < 0.001, **p < 0.01, *p < 0.05, and ns indicates no significance.

The presence of the blood-spinal cord barrier significantly restricts the delivery and utilization of many blood-borne therapeutic drugs [47]. Macrophage membrane-derived nanovesicles exhibit biomimetic properties that enable them to carry drugs across the BSCB and accumulate specifically at inflammatory sites. Furthermore, a growing body of evidence indicates that nanoparticles coated with macrophage membranes (MM) possess immune evasion capabilities, allowing them to effectively avoid phagocytic clearance by macrophages [48,49]. In our study, we leveraged these beneficial features to successfully deliver DHM across the BSCB, achieving effective drug accumulation at the site of injury (Fig. 3 A). To evaluate the targeting efficiency of MM@DHM, we performed in vivo fluorescence imaging after intravenous administration of CY5.5-labeled MM@DHM, MM@PLGA, and plain PLGA nanoparticles. Pronounced accumulation at the injury site was observed in the MM@DHM and MM@PLGA groups at 1, 3, and 7 days post-injection, whereas no significant signal was detected in the PLGA group (Fig. 3B and C). Ex vivo imaging of harvested tissues further corroborated these observations (Fig. 3D and E). Additionally, immunofluorescence staining of spinal cord sections demonstrated markedly enhanced accumulation of MM@DHM at the lesion site compared to uncoated PLGA nanoparticles (Fig. 3F). These biomimetic nanoparticles not only retain the advantageous physical properties of conventional nanoparticles but also inherit biological functions from their source cells, including high biocompatibility [50]. Histological examination via H&E staining revealed no detectable morphological alterations or tissue damage in any nanoparticle-treated group compared with sham controls (Supplementary Fig. S2 A). Subsequently, we evaluated the biodistribution of the nanoparticles in major organs (heart, liver, spleen, lungs, and kidneys, supplementary Fig. S2 B). Pronounced fluorescent signals were mostly observed in the liver and kidneys across all experimental groups at 3 days post injection, indicating these organs serve as primary clearance pathways. Furthermore, at 14 days post injection, negligible fluorescent signals were detected in all major organs, suggesting no abnormal long-term retention of the nanoparticles in vivo and preliminarily indicating their favorable biosafety. Furthermore, serum biochemical analysis demonstrated no marked differences in markers of liver function (ALT, AST) and renal function (BUN, UA) among the groups (Supplementary Fig. S 2C-F), confirming the absence of hepatorenal toxicity. Taken together, these findings indicate that MM@DHM exhibits excellent targeting efficiency and favorable biocompatibility, supporting its potential for therapeutic use.

### 3.4 Mitochondrial targeting and protecting effects of MM@DHM in vitro

**Fig. 4:**
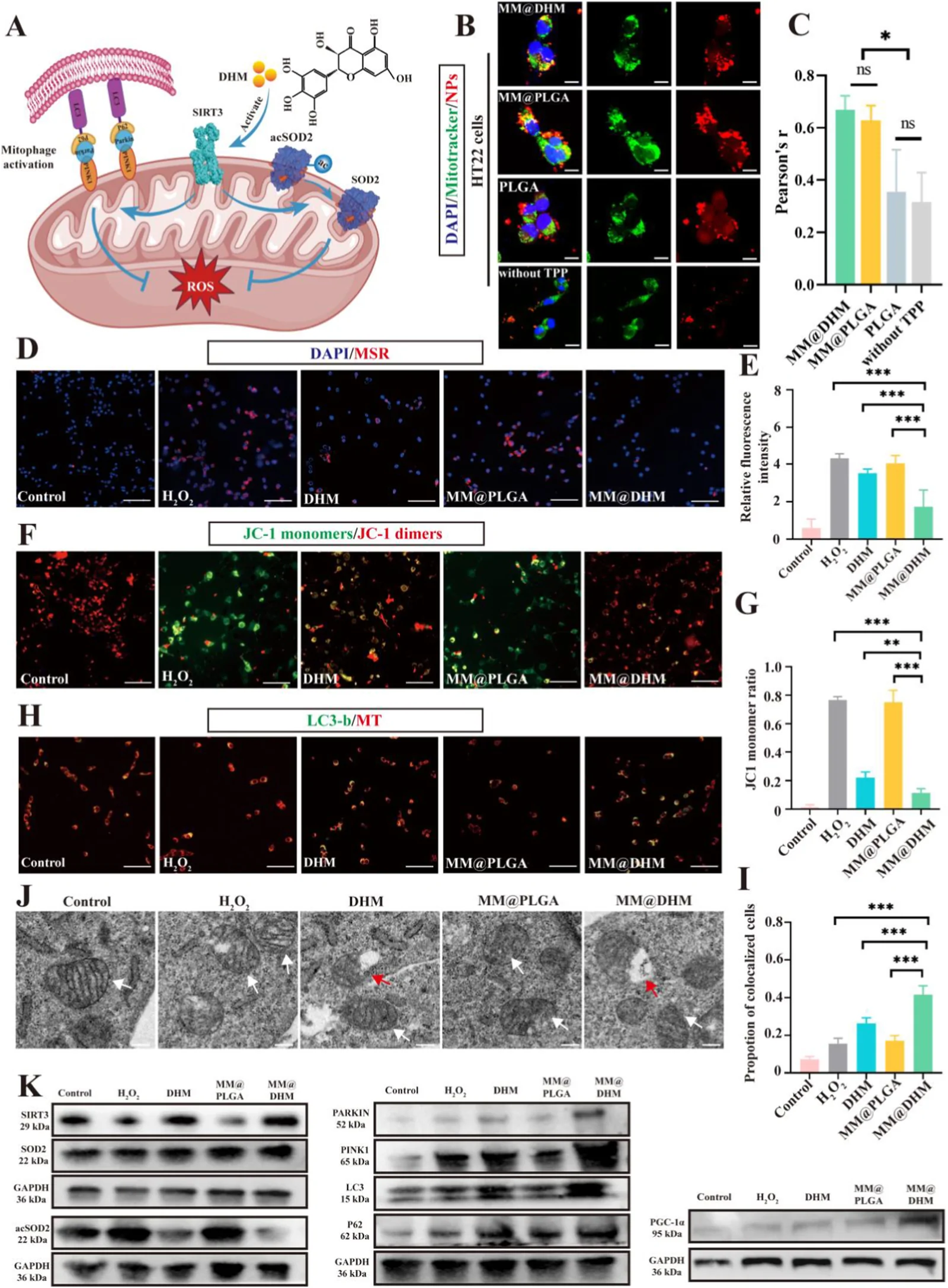
Mitochondrial targeting and mitochondrial protecting ability of MM@DHM. (A) Graphic demonstration of SIRT3 exerting its anti-ROS effect via SOD2 deacetylation and mitophage. (B) Representative fluorescence images showing co-localization between NPs and mitochondria from MM@DHM or PLGA treated HT22 cell (scale bar: 25 μm). (C) Quantification of the Pearson's r coefficient for NP-mitochondria colocalization (n = 3, calculated by JACoP plugin in imageJ). (D) Representative DAPI and MitoSox Red immunofluorescence staining of HT22 cells from different treatment groups. (scale bar: 100 μm); (E) Quantification of MSR signal from different treatment groups (n = 5). (F) Representative fluorescence microscopy images of HT 22 cells stained with JC-1 from different treatment groups (scale bar: 100 μm); (G) and quantification of JC-1 monomer ratio from different treatment groups (n = 5). (H) Representative LC3b and Mitotracker immunofluorescence staining of HT22 cells from different treatment groups. (scale bar: 100 μm); (I) Quantification of percentage of LC3b and Mitotracker double positive cells from different treatment groups (n = 5). (J) Representative TEM images showing mitochondria of HT22 cells from different treatment groups. (scale bar: 250 nm, white arrows indicated normal mitochondria, red arrows signified mitochondria undergoing mitophagy); (K) Western blot analysis of SIRT3 and its downstream protein expression in HT22 cells from different treatment groups. Data shown in C, E, G, I are presented as the mean ± standard error of the mean. ***p < 0.005, **p < 0.01, *p < 0.05, and ns indicates no significance. (For interpretation of the references to colour in this figure legend, the reader is referred to the Web version of this article.)

Disruption of the mitochondrial function leads to excessive generation of ROS, including hydroxyl radicals (∙OH) and superoxide anions (O_2_∙^-^), resulting in redox imbalance and subsequent cell death [51]. Based on such facts, our mitochondrial targeting strategy aims to enhance DHM-mediated SIRT3 activation, thereby initiating a dual endogenous anti-ROS mechanism: increasing SOD enzyme activity while facilitating mitophagy, and ultimately alleviating cellular oxidative stress. (Fig. 4 A). We initially examined the mitochondrial targeting capacity of MM@DHM in vitro. HT22 and BV2 cells were incubated with MM@DHM, MM@PLGA, PLGA or “without TPP” group (namely, “macrophage membrane coated DHM without TPP modification”) followed by immunofluorescence staining. The results demonstrated that both MM@DHM and MM@PLGA displayed markedly higher colocalization with MitoTracker relative to the PLGA or “without TPP″ group verifying that the encapsulation of TPP-modified MM significantly potentiates the mitochondrial targeting efficiency of the nano-drug delivery platform (Fig. 4B and C; Supplementary Fig. S4 A-B for BV2 cells). And mitochondrial ROS levels were assessed using MitoSOX Red staining across different treatment groups. Both DHM and MM@DHM significantly reduced mitochondrial ROS accumulation, with the nanoformulation demonstrating superior scavenging capacity compared to free DHM (Fig. 4D and E). Moreover, JC-1 staining and flow cytometry showed that treatment with MM@DHM effectively prevented the H_2_O_2_-induced loss of ΔΨm, suggesting protection of mitochondrial membrane integrity (Fig. 4F and G; Supplementary Fig. S3A). To evaluate mitophagy, we examined microtubule-associated protein 1 light chain 3B (LC3B), a central mediator of autophagosome formation in mammalian cells [52]. Quantitative analysis of MitoTracker-LC3B colocalization showed a significant increase from 15.52% in H_2_O_2_-treated cells to 26.21% (DHM) and 41.61% (MM@DHM), indicating enhanced mitophagy following nanoparticle treatment (Fig. 4H and I). Transmission electron microscopy further provided ultrastructural evidence of mitophagy in HT22 cells treated with DHM and MM@DHM (Fig. 4 J). Western blot analysis (Fig. 4 K) revealed upregulation of SIRT3 expression after both DHM and MM@DHM treatments, with a more pronounced effect in the nanoformulation group. Although total SOD2 protein levels remained unchanged, a decrease in acetylated SOD2 (acSOD2) indicated SIRT3-mediated deacetylation and subsequent activation of SOD2's antioxidant function. Furthermore, the study identified activation of the PINK1-Parkin pathway, which is known to mediate ubiquitination of mitochondrial outer membrane proteins for recognition by LC3B and autophagosome formation [53]. SIRT3 has been implicated in the regulation of PINK1/Parkin-mediated mitophagy [54]. Consistently, treated groups showed upregulation of proteins in the PINK1/Parkin-LC3B axis and concurrent change of p62, indicating enhanced mitophagic flux. Additionally, MM@DHM treatment also increased the expression of PGC-1α, suggesting the formation of a positive feedback loop within the SIRT3 signaling pathway. These findings indicate that MM@DHM help maintain mitochondrial homeostasis through SIRT3-mediated SOD2 activation and coordinated promotion of mitophagy pathways.

### 3.5 Mitigation of oxidative stress and inflammation by MM@DHM

**Fig. 5:**
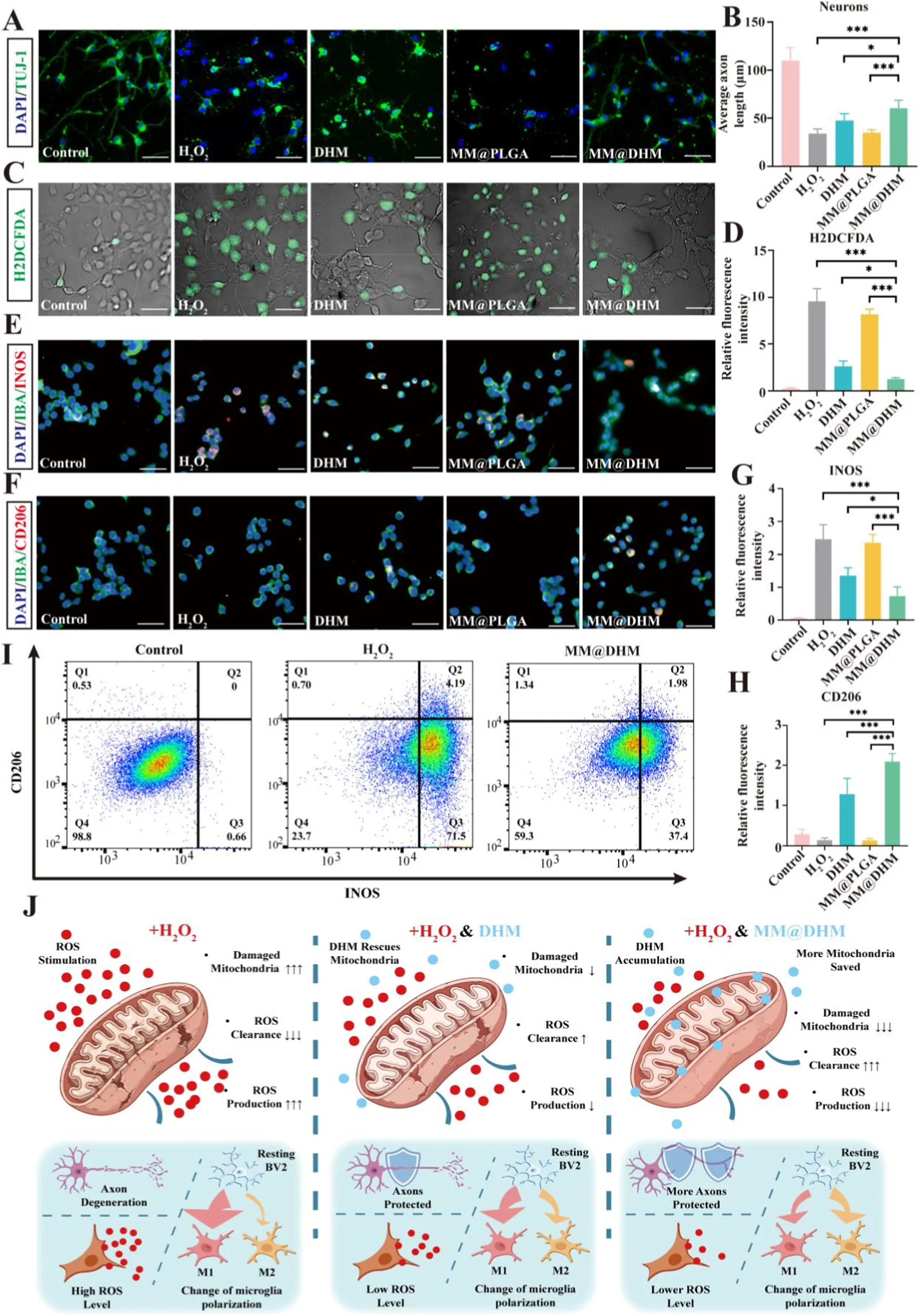
Antioxidant and anti-inflammation effects of MM@DHM in vitro. (A) Representative DAPI and TUJ-1 immunofluorescence staining of primary cortical neuron from different treatment groups. (scale bar: 50 μm); (B) Quantification of TUJ-1^+^ axon length from different treatment groups (n = 5; with at least 20 axons analyzed per replicate). (C) Representative fluorescence microscopy images of HT 22 cells stained with H_2_DCFDA from different treatment groups (scale bar: 50 μm); (D) Quantification of H_2_DCFDA ^+^ signal from different treatment groups (n = 5). (E) Representative DAPI, IBA-1 and INOS immunofluorescence staining of BV2 cells from different treatment groups. (scale bar: 50 μm); (F) Representative DAPI, IBA-1 and CD206 immunofluorescence staining of BV2 cells from different treatment groups. (scale bar: 50 μm); (G) Quantification of INOS^+^ signal from different treatment groups (n = 5). (H) Quantification of CD206^+^ signal from different treatment groups (n = 5). (I) Representative flow cytometry plots of INOS^+^ and CD206^+^ cells from different treatment groups. (J) Summary of MM@DHM targeting mitochondria to eliminate damaged mitochondria and intracellular ROS, thereby alleviating inflammation and promoting axonal regeneration; Data shown in B, D, G, H are presented as the mean ± standard error of the mean. ***P < 0.001, **p < 0.01, *p < 0.05, and ns indicates no significance.

To examine the effect of the nanoparticles on axonal regeneration under oxidative stress, primary cortical neurons were treated with H_2_O_2_ to establish an in vitro injury model. Results show that H_2_O_2_ exposure reduced the average axonal length to approximately 33 μm, while the average axon length of cortical neurons treated with MM@PLGA without DHM was 35 μm, and treatment with DHM and MM@DHM restored axonal lengths to 47 μm and 60 μm, respectively (Fig. 5A and B), suggesting that antioxidant intervention and metabolic support contribute to axonal protection. For ROS detection, HT22 cells under H_2_O_2_-induced oxidative stress were stained with H2DCFDA. Both DHM and MM@DHM significantly reduced intracellular ROS levels compared to the H_2_O_2_ group, with MM@DHM showing more pronounced efficacy, whereas MM@PLGA failed to reduce intracellular ROS levels (Fig. 5C and D). Live/dead staining and flow cytometry further indicated a reduction in cell death following DHM and MM@DHM treatment, with the nanoparticle group exhibiting the strongest protective effect against oxidative stress-induced apoptosis whereas MM@PLGA didn't**.** (Supplementary Fig. S3 B-C). During the secondary phase of SCI, locally accumulated ROS can exacerbate neuroinflammation by activating microglia and promoting their polarization toward a pro-inflammatory phenotype [55,56]. The BV2 microglial cell line is widely used as an in vitro model for neuroinflammatory studies [57]. In our experiments, BV2 cells were treated with H_2_O_2_ to mimic the oxidative microenvironment, while INOS and CD206 were used as markers for pro-inflammatory and anti-inflammatory phenotypes, respectively. Immunofluorescence and flow cytometry analysis revealed that, compared to the H_2_O_2_ group, both DHM and MM@DHM significantly decreased INOS expression and increased CD206 fluorescence intensity. Moreover, MM@DHM demonstrated a stronger ability than free DHM to modulate BV2 polarization, and MM@PLGA did not possess the aforementioned effects (Fig. 5E–I, Supplementary Fig. S4 D-G). These results suggest that MM@DHM effectively promotes a shift in microgl polarization from pro-inflammatory to anti-inflammatory states, likely attributable to its dual capacity to scavenge excess ROS and ameliorate mitochondrial dysfunction(Fig. 5 J).

### 3.6 Neuroprotective effects of MM@DHM in the acute phase of SCI

**Fig. 6:**
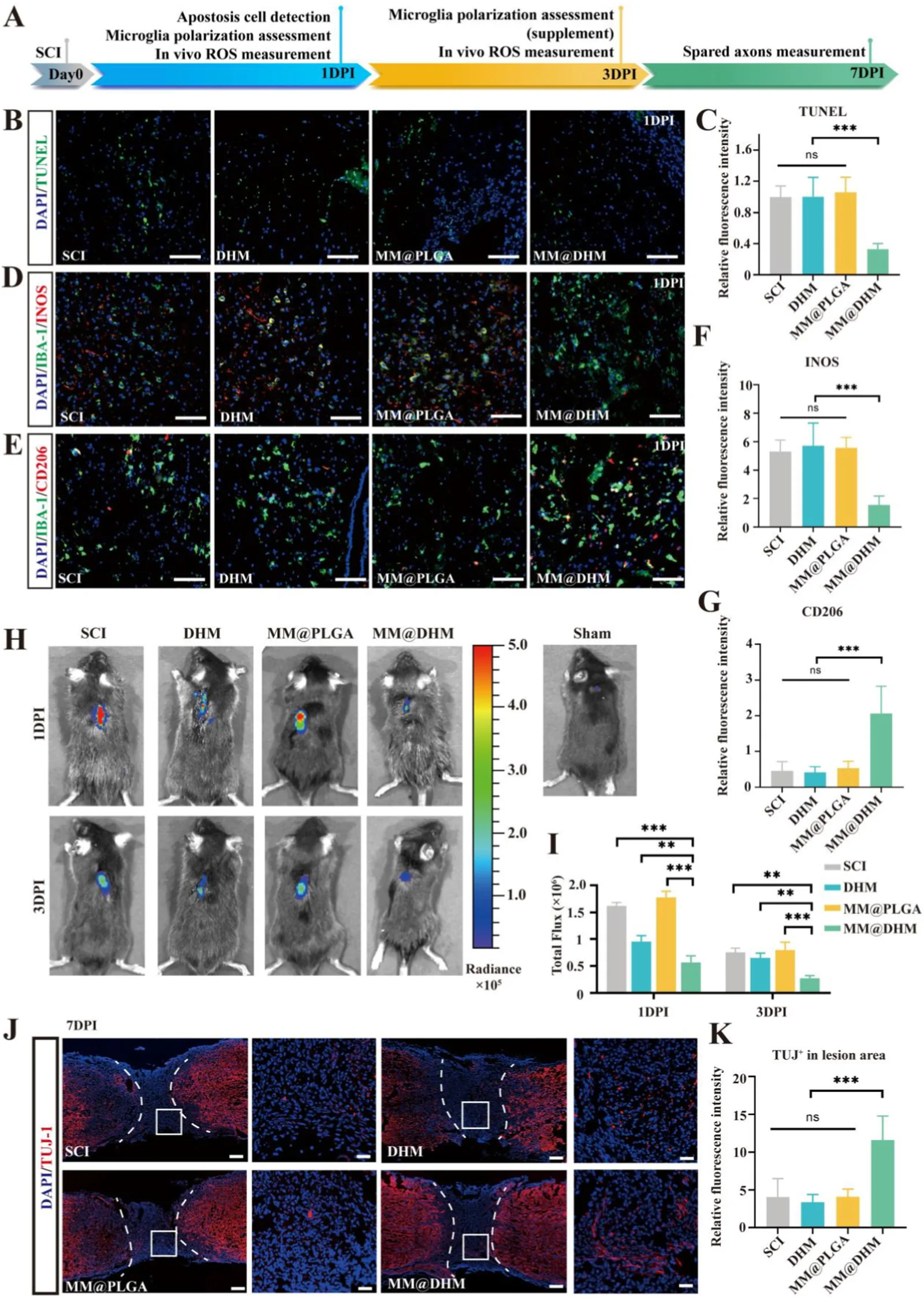
Neuroprotective effect of MM@DHM in acute and sub-acute SCI mice. (A) Experimental design for assessing the therapeutic effect of MM@DHM in mice with acute and sub-acute SCI; (B) Representative DAPI and TUNEL immunofluorescence staining of the lesion site in different treatment groups at 1 DPI. (scale bar: 100 μm); (C) Quantification of TUNEL^+^ signal at the lesion site from different treatment groups at 1 DPI (n = 5); (D) Representative DAPI, IBA-1 and INOS immunofluorescence staining of the lesion site in different treatment groups at 1 DPI. (scale bar: 100 μm); (E) Representative DAPI, IBA-1and CD206 immunofluorescence staining of the lesion site in different treatment groups at 1 DPI. (scale bar: 100 μm); (F) Quantification of INOS^+^ signal of the lesion site in different treatment groups at 1 DPI (n = 5); (G) Quantification CD206^+^ signal f the lesion site in different treatment groups at 1 DPI (n = 5); (H) Representative in vivo bioluminescence images of L-012-stained oxidative stress in mice receiving different treatments at different time point; (I) Quantification of total flux (p/s) from different treated groups at different time point (n = 3); (J) Representative DAPI and TUJ-1 immunofluorescence staining of injured spinal cord from different treatment groups at 7 DPI. (scale bar: 200 μm; 40 μm, dash line indicated lesion border); (K) Quantification of TUJ-1^+^ signal in lesion site of injured spinal cord from different treatment groups at 7 DPI (n = 5). Data shown in C, F, G, I, K are presented as the mean ± standard error of the mean. ***P < 0.005, **p < 0.01, *p < 0.05, and ns indicates no significance.

Under oxidative stress, the collapse of mitochondrial membrane potential triggers the release of cytochrome C, ultimately leading to the formation of apoptotic vesicles and activation of pro-apoptotic factors such as caspase-3 and Bax, thereby inducing programmed cell death [58,59]. Following SCI, the intense oxidative stress and inflammatory response during the acute phase contribute to extensive axonal degeneration. To evaluate the neuroprotective effect of MM@DHM in the acute stage of injury, we conducted immunofluorescence staining on spinal cord tissues from all experimental groups at 1, 3, and 7 DPI (Fig. 6 A). At 1 DPI, MM@DHM treatment significantly reduced apoptosis around the lesion border compared to the SCI, DHM, and MM@PLGA groups (Fig. 6B and C). During the acute phase of SCI, activated microglia and infiltrating blood-derived macrophages predominantly adopt a pro-inflammatory phenotype, characterized by elevated expression of pro-inflammatory markers such as INOS and IL-1β [60,61]. In contrast, anti-inflammatory macrophages promote tissue repair through expression of anti-inflammatory markers including CD206 and ARG-1 [62,63]. To assess the immunomodulatory effect of MM@DHM, we analyzed spinal cord tissues at 1 and 3 DPI. At 1 DPI, MM@DHM treatment significantly increased the proportion of CD206^+^ anti-inflammatory microglia/macrophages while reducing the number of iNOS^+^ pro-inflammatory cells compared to the SCI, DHM, and MM@PLGA groups (Fig. 6D–G), corresponding trends were also observed for IL-1β and Arg-1 expression (Supplementary Fig. S5A-B, E-H), further supporting the anti-inflammatory effect of MM@DHM. Similar results were observed at 3 DPI, where MM@DHM consistently elevated CD206 expression and decreased iNOS expression relative to control groups (Supplementary Fig. S5C-D, G-H). Consistently, Western blotting and ELISA on spinal cord homogenates at 1 DPI further confirmed that MM@DHM significantly upregulated CD206 protein levels and downregulated iNOS protein levels compared to the SCI, DHM, and MM@PLGA groups (Supplementary Fig. S5 I-K). Furthermore, in vivo ROS detection using L012 chemiluminescence showed a sharp peak in ROS levels at 1 DPI, which was markedly suppressed by MM@DHM treatment (Fig. 6H and I). Notably, administration of MM@DHM also preserved a greater number of Tuj-1^+^ neurons in the lesion core (Fig. 6J and K), an effect likely attributable to its dual ability to modulate the injury microenvironment and provide metabolic support for axonal regeneration. Together, these findings demonstrate that mitochondria-targeted nanoparticles effectively alleviate oxidative damage, attenuate neuroinflammation, and promote axonal repair during the acute phase after SCI.

### 3.7 RNA sequencing reveals the therapeutic mechanisms of MM@DHM

**Fig. 7:**
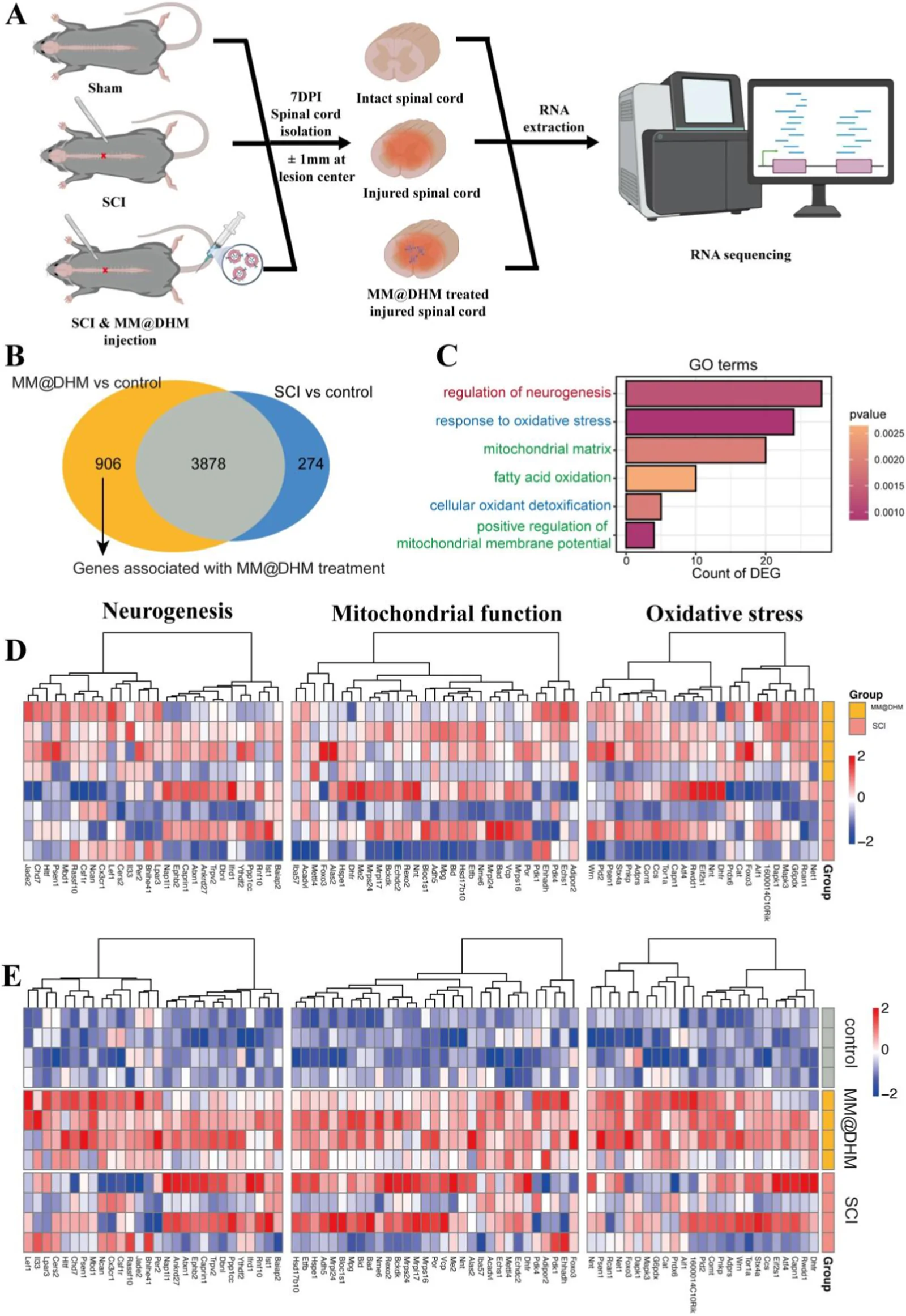
RNA-seq revealed the mechanism of therapeutic effect of MM@DHM. (A) Graphic demonstration showing acquisition process of samples for RNA-seq; (B) Venn diagram of DEGs among control (sham), SCI and MM@DHM groups; (C) GO enrichment analysis of DEGs between MM@DHM and control groups; (D) Heatmap of DEGs associated with MM@DHM treatment between SCI and MM@DHM groups; (E) Heatmap of DEGs associated with MM@DHM treatment among control, SCI and MM@DHM groups.

To elucidate the molecular mechanisms underlying the therapeutic effects of MM@DHM in SCI, mice were divided into three groups: control, DHM, and MM@DHM. Seven days post-injury and nanoparticle administration, tissues surrounding the lesion site were harvested for reference-guided RNA sequencing (Fig. 7 A). Gene Ontology (GO) analysis identified 4784 differentially expressed genes (DEGs) in the MM@DHM group compared to SCI controls, among which 906 genes were specifically regulated by MM@DHM treatment (Fig. 7 B). Functional annotation revealed that MM@DHM significantly upregulated 28 neurogenesis-related genes (e.g., Ankrd27, Atxn1), 34 genes associated with mitochondrial function—including 4 regulators of membrane potential (Bad, Bid, Nnt, Vcp), 20 mitochondrial matrix genes (Acadvl, Alas2, etc.), and 10 fatty acid oxidation genes (Acadvl, Adh5, etc.)—as well as 29 oxidative stress-related genes, comprising 24 involved in oxidative stress response (1600014C10Rik, Adprs, etc.) and 5 related to cellular oxidative repair (Cat, Ccs, Dhfr, Nnt, Prdx6) (Fig. 7C–E).These transcriptomic results demonstrate that MM@DHM not only effectively alleviates oxidative damage and mitochondrial dysfunction but also significantly contributes to neural circuit regeneration and remodeling. The gene expression profile highlights the multi-target therapeutic potential of this mitochondria-targeted nanodrug in the treatment of SCI.

### 3.8 MM@DHM promotes neural regeneration in chronic SCI phase

**Fig. 8:**
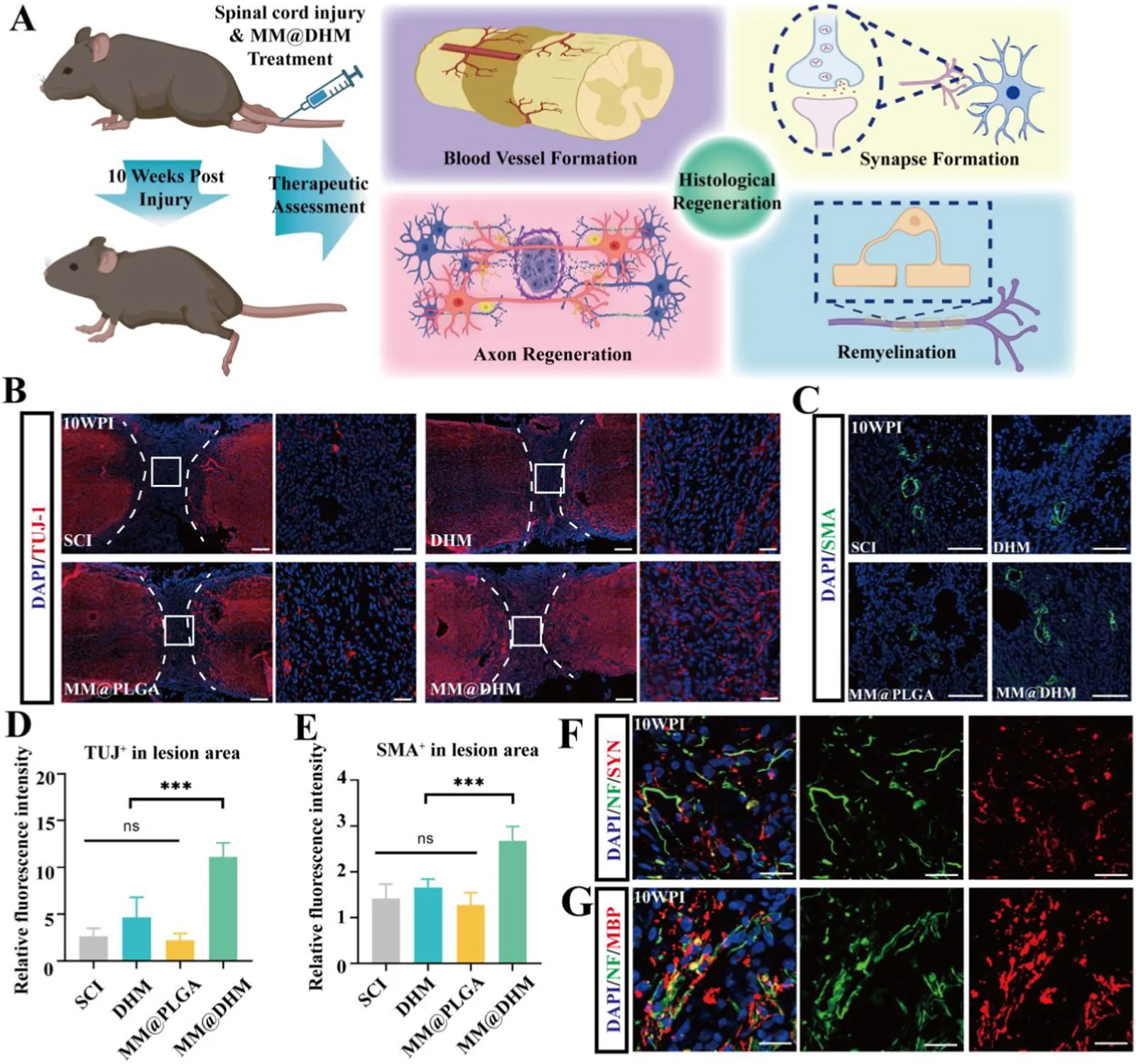
The long-term histological regenerative effect of MM@DHM. (A) Long-term histological detection workflow schematic after SCI; (B) Representative DAPI and TUJ-1 immunofluorescence staining of injured spinal cord from different treatment groups at 10 weeks post-injury (WPI). (scale bar: 200 μm; 40 μm); (C) Representative DAPI and SMA immunofluorescence staining of the lesion site in different treatment groups at 10 WPI. (scale bar: 50 μm, dash line indicated lesion border); (D) Quantification of regenerated TUJ-1^+^ axons at the lesion site in different treatment groups (n = 5); (E) Quantification of regenerated SMA^+^ blood vessels (n = 5) (B) at the lesion site in different treatment groups; (F) Representative DAPI, NF and SYN immunofluorescence staining of the lesion site from MM@DHM treated mice at 10 WPI. (scale bar: 25 μm); (G) Representative DAPI, NF and MBP immunofluorescence staining of the lesion site from MM@DHM treated mice at 10 WPI. (scale bar: 25 μm). Data shown in D, E are presented as the mean ± standard error of the mean. ***P < 0.005, and ns indicates no significance.

To evaluate the long-term therapeutic effects of MM@DHM, we conducted a series of experiments during the chronic phase of SCI (Fig. 8 A). Age- and gender-matched mice were assigned to four groups: untreated control, DHM, MM@PLGA, and MM@DHM. 10 weeks post-injury, spinal cord tissues were collected and analyzed using immunofluorescence staining. The results revealed a significant increase in TUJ-1^+^ cells within the lesion core in the MM@DHM group compared to the other treatments, indicating enhanced axonal regeneration (Fig. 8B–D). In addition, immunofluorescence analysis revealed a significant increase in 5-hydroxytryptamine (5-HT^+^) signals, a marker specific to descending projection tracts, within the lesion area of the MM@DHM group compared to other groups. Although the effect was less pronounced than the overall axonal response reflected by TUJ-1 staining, it still suggests that MM@DHM provides certain supportive effects on the repair of descending nerve fiber tracts after SCI (Fig. S5 A-B). Histological assessment via H&E staining showed that MM@DHM treatment improved tissue integrity and continuity at the injury site, accompanied by a reduction in lesion cavity formation relative to control groups (Supplementary Fig. 5C). Furthermore, enhanced angiogenesis was observed in peri-lesion regions following MM@DHM administration, suggesting facilitation of vascular recovery and blood supply restoration (Fig. 8C–E). Immunofluorescence analysis demonstrated strong colocalization of neurofilament (NF, a marker of mature axons) with synaptophysin (SYN, a synaptic marker) in MM@DHM-treated mice, indicating promoted synaptic reformation (Fig. 8F). Increased colocalization of NF with myelin basic protein (MBP) further confirmed enhanced axonal remyelination in the MM@DHM group (Fig. 8 G), a critical process for restoring efficient signal conduction in neural circuits [[64], [65], [66]].Together, these findings indicate that early intervention with MM@DHM to alleviate oxidative stress damage leads to significant improvements in long-term neural regeneration during chronic SCI. The observed therapeutic benefits—including structural preservation, vascular remodeling, synaptic reorganization, and functional remyelination—collectively underscore the comprehensive neurorestorative potential of this mitochondrial-targeted nanodrug.

### 3.9 MM@DHM promotes long-term sensorimotor functional recovery after SCI

**Fig. 9:**
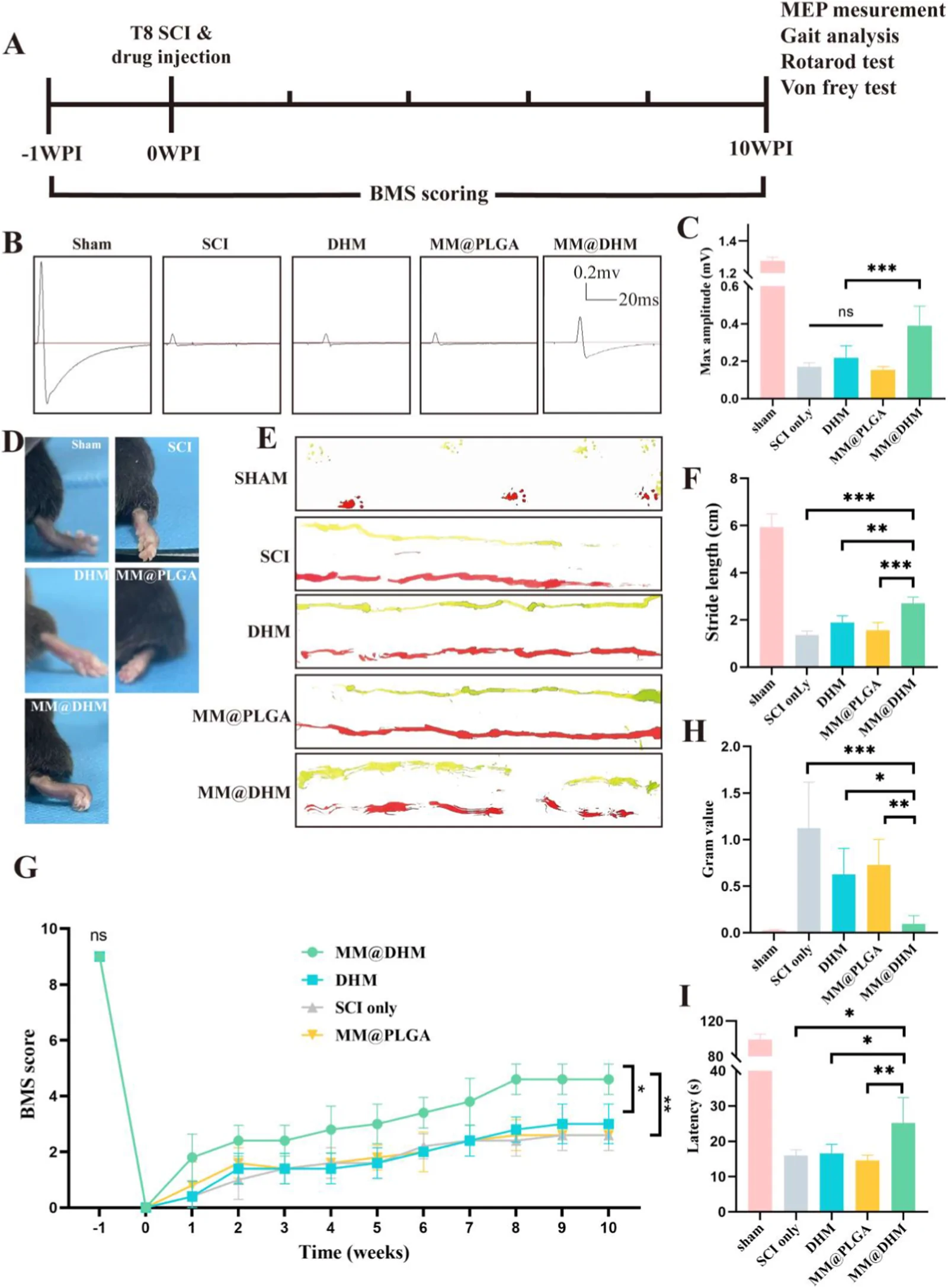
MM@DHM promotes long-term sensorimotor functional recovery after SCI. (A) Experimental design of MM@DHM treatment and behaviour assessment; (B) Representative motor evoked potential (MEP) profiles of mice in different treatment group at 10 WPI; (C) Quantification of MEP amplitude in different treatment groups at 10 WPI; (D) Representative leg images from different treatment groups at 10 WPI; (E) Representative footprint images of different groups at 10 WPI; (F) Quantification of stride length in different treatment groups during gait analysis at 10WPI; (G) BMS scores of mice from different groups from 1 to 10 WPI; (H) Quantification of gram value in different treatment groups during Von-frey test at 10WPI; (I) Quantification of latency to fall in different treatment groups during rotarod test at 10WPI. Data shown in D-G and I are presented as the mean ± standard error of the mean. ***P < 0.005, **p < 0.01, *p < 0.05, and ns indicates no significance; (n = 5 in B, C, F,G, H, I) BMS scores were compared using Mann-Whitney *U* test.

To evaluate the long-term therapeutic efficacy of MM@DHM, we performed comprehensive sensorimotor functional assessments across all experimental groups (Fig. 9 A). Hindlimb motor function evaluated using the Basso Mouse Scale (BMS) revealed that the MM@DHM group achieved significantly higher average scores (approaching 5 points) at 10 weeks post-injury (WPI) [67], compared to the DHM, SCI-only, and MM@PLGA groups (which scored between 2 and 3 points) (Fig. 9G). Mice treated with MM@DHM also exhibited superior hindlimb weight-bearing capacity (Fig. 9D). Electrophysiological measurements conducted at 10 weeks showed that motor-evoked potentials in the MM@DHM group reached amplitudes of approximately 0.4 mV—still below sham levels (1.2 mV) but significantly higher than those in other treatment groups—indicating partial recovery of neural conduction (Fig. 9B and C). Gait analysis demonstrated increased stride length in MM@DHM-treated mice compared to controls (Fig. 9E and F), along with improved performance on the rotarod test (Fig. 9I). Sensory evaluation indicated a near-normalization of pain threshold in the MM@DHM group (average force: 0.096 g), closely approaching sham levels (0.028 g) (Fig. 9H).These functional improvements correlate with the neural regeneration observed at the lesion site, demonstrating that MM@DHM treatment effectively facilitates both motor and sensory recovery after SCI. The multi-modal nature of these gains suggests comprehensive restoration of neural circuits involved in hindlimb function.

## 4 Discussion

Oxidative stress and mitochondrial dysfunction following SCI are critical factors that impede neural regeneration, highlighting the therapeutic importance of mitochondrial-targeted strategies. SIRT3, a key molecule in maintaining mitochondrial homeostasis, participates in numerous cellular metabolic processes [68,69], and oxidative stress injury has been associated with reduced SIRT3 activity [70]. In this study, we first observed a significant downregulation of SIRT3 expression in the local injured area after SCI. Targeting this key pathological event, we successfully constructed a biomimetic cascade-targeting nanoplatform, MM@DHM. Utilizing a macrophage membrane biomimetic camouflage strategy, the nanoplatform achieves active homing to the lesion site through interactions between membrane surface adhesion molecules (such as Integrin-α4/Mac-1) and VCAM-1 highly expressed on inflamed endothelial cells [34]. Subsequent TPP cation-mediated mitochondrial targeting enables the therapeutic agent to traverse the cytoplasmic barrier and directly reach the primary subcellular localization of SIRT3. By precisely delivering the natural small-molecule agonist DHM to mitochondria, MM@DHM achieves efficient SIRT3 reactivation, alleviates early ROS accumulation, mitigates local neuroinflammation, preserves greater axonal fibers, and ultimately promotes sensorimotor functional recovery in mice. This cascade-targeting design overcomes the limitations of conventional administration routes, including low bioavailability and pronounced off-target effects. Moreover, the use of biologically derived membrane components enhances the biocompatibility of the nanoplatform with the host environment.

This study not only provides a novel targeted therapeutic strategy for SCI but also, through in-depth mechanistic dissection of this platform, offers new experimental evidence for understanding the central role of SIRT3 in post-traumatic redox homeostasis regulation. Our results reveal a SIRT3-driven positive feedback regulatory loop: MM@DHM-mediated SIRT3 activation not only directly enhances SOD2 activity to scavenge the early burst of ROS but, more importantly, triggers downstream upregulation of PGC-1α. Given that PGC-1α itself is a key upstream regulator of SIRT3 transcription (via the NRF-1/2 pathway) [71,72], this finding suggests that exogenous restoration of SIRT3 activity can activate the impaired PGC-1α/SIRT3 axis, thereby amplifying its antioxidant and mitochondrial bioenergetic effects, which is consistent with our observation of increased mitophagic flux. The discovery of this positive feedback loop provides theoretical support for explaining why SIRT3 agonists can exert significant therapeutic efficacy even under conditions of low baseline expression. Furthermore, as the primary mitochondrial deacetylase, the functional complexity of SIRT3 extends far beyond mere ROS scavenging. For instance, in ischemia-reperfusion injury, SIRT3 inhibits mPTP opening through deacetylation of CypD and p53, directly blocking apoptotic signaling [73]; whereas in tumor cells, it reshapes energy metabolism by modulating HIF-1α stability [74]. Integrating these findings, we speculate that the neuroprotective effects of MM@DHM are multidimensional, with its essence lying in the precise delivery of a homeostatic regulator to the core of the lesion, thereby exerting more comprehensive therapeutic effects than a single antioxidant agent.

Several limitations of this study warrant further discussion. First, although biosafety has been systematically evaluated, the long-term biodistribution, degradation pathways, and potential immunogenicity of biomimetic nanomedicines require further investigation. Second, regarding the SIRT3/PGC-1α interaction network, although our study found that MM@DHM also modulates PGC-1α expression, current data cannot distinguish whether this is a direct feedback effect or mediated through other intermediate molecules (such as AMPK or NAD^+^ levels). Further studies are warranted to elucidate this complex regulatory network. Third, although our results demonstrate that MM@DHM exerts significant pro-neural repair effects, whether the observed therapeutic outcomes are primarily attributable to neuroprotection or neuroregeneration remains to be further elucidated. Retrograde tract-tracing experiments will be of great value in definitively distinguishing between these two possibilities. Finally, as a biomimetic nanoplatform derived from natural cell membranes, membrane proteins may lose integrity or bioactivity during long-term storage, potentially compromising the targeting capability and biological functions of the nanoparticles. In addition, batch-to-batch variability in membrane coating efficiency remains a concern, as subtle differences in membrane extraction and fusion processes could lead to inconsistent nanoparticle properties. Standardized manufacturing protocols and rigorous quality control measures are essential for future large-scale production and clinical translation.

In summary, this study developed a biomimetic nanoplatform, MM@DHM, and provided robust evidence for the significance of targeted SIRT3 activation in re-establishing redox homeostasis and promoting neural regeneration following SCI. We propose a cascade-targeting drug delivery strategy centered on functional restoration of specific pathological organelles. This strategy may also be generalizable to other neurological disorders characterized by mitochondrial dysfunction and oxidative stress, such as traumatic brain injury and neurodegenerative diseases. This study provides a compelling proof-of-concept for clinical translation and demonstrates a reliable delivery vehicle with promising application prospects.

## 5 Conclusion

In this study, a macrophage membrane-camouflaged biomimetic drug delivery system (MM@DHM) was developed to efficiently deliver the natural flavonoid compound DHM to the mitochondria of key effector cells in the injured area for the treatment of SCI. The macrophage membrane coating provided the system with excellent biocompatibility and inflammatory tropism, significantly enhancing the accumulation of DHM at the injury site, while TPP modification conferred superior mitochondrial targeting ability. We found that MM@DHM alleviated oxidative stress damage by enhancing SOD2 enzyme activity and activating mitophagy. This nanotherapeutic agent effectively promoted axonal regeneration and suppressed inflammatory responses, thereby contributing to the recovery of neurological function after SCI. Collectively, these findings demonstrate that integrating bioactive macrophage membranes modified with targeting molecules with suitable polymeric nanoparticles may offer a promising strategy for the treatment of SCI.

## CRediT authorship contribution statement

**Yu Zhou:** Conceptualization, Data curation, Formal analysis, Investigation, Methodology, Project administration, Resources, Software, Supervision, Validation, Visualization, Writing – original draft. **Yijie Kong:** Conceptualization, Data curation, Formal analysis, Investigation, Methodology, Project administration, Resources, Validation, Visualization. **Zhiquan Yang:** Software, Supervision. **Kai Zhang:** Investigation, Methodology. **Runlin Wen:** Data curation, Formal analysis. **Shiyu Fu:** Investigation, Supervision. **Wanrong Ma:** Project administration, Validation. **Ge Long:** Conceptualization, Funding acquisition, Supervision, Validation, Writing – review & editing. **Xing Li:** Data curation, Funding acquisition, Validation, Writing – review & editing. **Dingyang Liu:** Conceptualization, Data curation, Formal analysis, Funding acquisition, Writing – review & editing.

## Declaration of competing interests

The authors declare that they have no known competing financial interests or personal relationships that could have appeared to influence the work reported in this paper.

## Data Availability

Data will be made available on request.
