# Supplementary material for: Hierarchical targeting biomimetic nanoparticles directing to lesion site and mitochondria promote neuroregeneration and attenuate inflammation after spinal cord injury
Source: Mater Today Bio. 2026 Aug 12;40:103544. doi: 10.1016/j.mtbio.2026.103544 (PMC13521229; doi:10.1016/j.mtbio.2026.103544)
Supplement: Supplementary file 1 [file mmc1.docx]

**Supplementary materials**

**Figure S1 Immunofluorescence co-localization analysis of SIRT3.**

(A) Representative immunofluorescence images of SIRT3 and neuronal marker NEUN in uninjured and 1-day post-injury (1 DPI) spinal cord sections (scale bar: 200 μm, split channels).

(B) Representative immunofluorescence images of SIRT3 and pan-microglial marker IBA-1 in uninjured and 1 DPI spinal cord sections (scale bar: 200 μm, split channels).

(C) Representative immunofluorescence images showing co-localization of SIRT3 with M1-type microglia (INOS) at 1 DPI. White arrows indicate cells co-expressing SIRT3 and M1 phenotype (scale bar: 25 μm).

(D) Representative immunofluorescence images showing co-localization of SIRT3 with M2-type microglia (CD206) at 1 DPI. White arrows indicate cells co-expressing SIRT3 and M2 phenotype (scale bar: 25 μm).

**Figure S2 MM@DHM has good biocompatibility**

(A) Representative images of hematoxylin and eosin-stained sections of the major organs from different-treated 3DPI mice (scale bar: 100 μm).

(B) Representative ex vivo fluorescence images of main organs (from left to right: heart, liver, speech, lung, kidney) dissected from 3DPI or 2WPI mice after intravenous injection with Cy5-labeled MM@DHM, MM@PLGA or PLGA.

(C-E) Hematology assay of different-treated 3DPI mice (n=3).

Data shown in C-E are presented as the mean ± standard error of the mean and ns indicates no significance.

**Figure S3 Therapeutic effect of MM@DHM on HT22 cells**

(A) Representative flow cytometry histograms of JC-1 monomer HT22 cells from different treatment groups (gating strategy listed below).

(B) Representative fluorescence microscopy images of HT 22 cells stained with Calcein/PI from different treatment groups (scale bar: 100 μm).

(C) Representative flow cytometry plots of Annexin V/PI staining of HT22 cells from different treatment groups (gating strategy listed below).

**Figure S4 Therapeutic effect of MM@DHM on BV2 cells**

(A) Representative fluorescence images showing co-localization between NPs and mitochondria from MM@DHM, MM@PLGA, PLGA or MM@DHM without TPP modification (without TPP) treated BV2 cell (scale bar: 25 μm).

(B) Quantification of the Pearson's r coefficient for NP-mitochondria colocalization (n=3, calculated by JACoP plugin in imageJ).

(C) Representative flow cytometry plots of INOS^+^ and CD206^+^ BV2 cells from different treatment groups (gating strategy listed below).

(D-G) Relative mRNA expression of polarization marker of BV2 cells from different treatment groups (n=3) (C: INOS; D: CD206; E: IL-1β; F: ARG-1).

**Figure S5 The effect of MM@DHM on microglia polarization**

(A-B) Representative DAPI, IBA-1 and IL-1β (A) or ARG-1 (B) immunofluorescence staining of the lesion site in different treatment groups at 1 DPI (scale bar: 100 μm).

(C-D) Representative DAPI, IBA-1 and INOS (A) or CD206 (B) immunofluorescence staining of the lesion site in different treatment groups at 3 DPI (scale bar: 100 μm).

(E-H) Quantification of IL-1β (E); ARG-1 (F); INOS (G) or CD206 (H) signal (n=5).

(I) Western blot analysis of INOS and CD206 protein expression in the spinal cord tissues from different treatment groups at 1 DPI.

(J-K) ELISA quantification of INOS (J) and CD206 (K) levels in the spinal cord tissues from different treatment groups at 1 DPI (n=3).

Data shown in E-H, J-K are presented as the mean ± standard error of the mean. ***P<0.005, **P<0.01 and ns indicates no significance.

**Figure S6** **The effect of MM@DHM on spinal cord structure and 5-HTergic nerve fiber regeneration at 10 weeks post-injury (WPI)**

(A) Representative DAPI and 5-HT immunofluorescence images of the injured spinal cord from different treatment groups at 10 WPI. Dashed lines indicate the lesion borders; white squares and the corresponding magnified images below show the lesion core area (scale bar: 200 μm, 40 μm).

(B) Quantification of relative 5-HT fluorescence intensity in the lesion core across different groups (n=5).

(C) Representative hematoxylin and eosin (H&E)-stained longitudinal sections of the spinal cord from different treatment groups at 10 WPI, showing histomorphological changes and glial scar formation (scale bar: 250 μm).

Data in B are presented as the mean ± standard error of the mean (SEM). *P < 0.05, **P < 0.01.
